# Comparative analysis of drying kinetics, diffusivity, and thermodynamic properties of hybrid solar and oven dryers for Egyptian sweet marjoram

**DOI:** 10.1038/s41598-025-14663-z

**Published:** 2025-08-19

**Authors:** El-Sayed G. Khater, Adel H. Bahnasawy, Awad Ali Tayoush Oraiath, Abdallah Elshawadfy Elwakeel, Ahmed Elbeltagi, Ali Salem, Ahmed Z. Dewidar, Abdelaziz M. Okasha, Khaled A. Metwally

**Affiliations:** 1 Agricultural and Biosystems Engineering Department, Faculty of Agriculture, Benha University, P.O. Box 13736, Moshtohor, Toukh, Kalubia Egypt; 2 Department of Agricultural Engineering, Faculty of Agriculture, Omar Al Mukhtar University, P.O. Box 991, Al Bayda, Libya; 3 Agricultural Engineering Department, Faculty of Agriculture and Natural resources, Aswan University, Aswan, Egypt; 4 Agricultural Engineering Department, Faculty of Agriculture, Mansoura University, Mansoura, 35516 Egypt; 5 Structural Diagnostics and Analysis Research Group, Faculty of Engineering and Information Technology, University of Pécs, Pécs, Hungary; 6 Prince Sultan Bin Abdulaziz International Prize for Water Chair, Prince Sultan Institute for Environmental, Water and Desert Research, King Saud University, Riyadh, 11451 Saudi Arabia; 7 Department of Agricultural Engineering, Faculty of Agriculture, Kafrelsheikh University, Kafr El-Sheikh, 33516 Egypt; 8 Soil and Water Sciences Department, Faculty of Technology and Development, Zagazig University, Zagazig, 44519 Egypt

**Keywords:** Herbs and spices, Solar drying, Renewable energy, Thin layer modeling, Gibbs free energy, Enthalpy and entropy, Plant sciences, Energy science and technology

## Abstract

Egyptian sweet marjoram leaves (ESML) are aromatic herbs long valued for their ability to enhance flavor and extend shelf life by inhibiting the autoxidation of food lipids. Despite their widespread use, limited research exists on how various drying techniques, air temperatures, and thin-layer thicknesses affect the drying behavior, mathematical modeling, effective moisture diffusivity (EMD), activation energy, and thermodynamic properties of ESML. This study addresses this gap by examining the drying characteristics of ESML using a hybrid solar drying system (HSDS) at three air temperatures (50, 60, and 70 °C) and three layer thicknesses (1, 2, and 3 cm), comparing with a conventional oven drying (OD). The findings revealed that HSDS at 70 °C with a 3 cm layer thickness achieved the highest equilibrium moisture content (EMC), while HSDS at the same temperature with a 1 cm layer thickness resulted in the shortest drying time, highest drying rate, and moisture ratio. Additionally, the highest activation energy was observed using HSDS at 70 °C and a 2 cm layer thickness. Notably, drying at 70 °C with a 1 cm layer thickness reduced drying time by 66.67% compared to drying at 50 °C for the same thickness. Nonlinear regression analysis of eleven thin-layer drying models identified Weibullian (I) and Midilli as the best-fitting models for HSDS and OD, respectively. The HSDS demonstrated comparable performance to the OD while utilizing solar energy as a renewable heat. These findings advance the knowledge of drying systems by demonstrating the effectiveness and sustainability of hybrid solar drying for preserving the quality and functional properties of medicinal and aromatic herbs like marjoram.

## Introduction

There is a burgeoning interest in the exploration of natural antioxidants extracted from plants, largely influenced by the global shift toward the utilization of natural additives in food and cosmetic products^1–3^. Among the various sources of such antioxidants, herbs and spices represent significant avenues for research, particularly in terms of safety^4,5^. Sweet marjoram, a widely recognized culinary herb, has a cultivation history in Egypt that spans over three millennia, with Egypt currently accounting for approximately 90% of the global supply of this herb^6^. In the realm of traditional folk medicine, sweet marjoram has been employed in the form of herbal tea (infusion) to address a range of health issues, including asthma, colds, coughs, cramps, depression, dizziness, gastrointestinal disorders, hay fever, headaches, toothaches, sinus congestion, as well as acting as a diuretic and promoting menstruation^7,8^. The herb is composed of various bioactive compounds, including carnosic, oleanolic, and ursolic acids; cis-sabinene hydrate; flavonoids such as diosmetin, luteolin, and apigenin; hydrocarbons including P-cymene and γ-terpinene; phenolic glycosides like arbutin, methyl arbutin, vitexin, orientin, and thymonin; phenolic terpenoids such as thymol and carvacrol; tannins; sitosterol; and triacontane^7–10^. Moreover, sweet marjoram leaves, particularly when consumed as herbal tea, may function as an immunostimulant and aid in diminishing genotoxicity in patients undergoing chemotherapeutic treatments^6^. Freshly harvested spices and herbs are characterized by high MC and the presence of various microorganisms, necessitating immediate preservation to prevent biological deterioration due to their perishable nature^11^. Thermal drying represents the most widely employed, cost-effective method of post-harvest processing, effectively minimizing losses of these raw materials^12,13^. Furthermore, this process is crucial for mitigating potential safety hazards associated with toxin formation^14,15^.

Drying is one of the most ancient methods of food preservation and serves a vital function within the food industry^16–18^. The primary objective of this process is to reduce MC and water activity to safe levels, thereby extending product shelf life, minimizing packaging requirements, and lowering shipping weights^19–21^. Consequently, drying techniques are extensively employed for the dehydration of various food items, including vegetables, fruits, spices, herbs, and other products^22–25^. In contemporary markets, there is an increasing demand among consumers for processed foods that retain the original characteristics of fresh produce^26,27^. Therefore, it is imperative that the drying process is executed with precision to preserve the flavor, aroma, color, appearance, and nutritional value of the plants to the greatest extent possible^28^. Moreover, drying efficiency is an essential metric for assessing drying performance. This encompasses factors such as energy consumption, drying time, and drying rate, all of which are critical for optimizing the drying process in both quality and operational efficiency^29^.

Solar energy represents one of the most extensively utilized renewable energy sources for the drying of a variety of products, including fruits^30,31^ vegetable^32–36^ fish^37–41^ spices and herbs^42–45^ etc. The process of solar drying is both economical and sustainable, offering an environmentally friendly approach for agricultural products^46–48^. Solar drying methods can be categorized based on the utilization of solar energy into three primary groups: (a) direct solar drying, in which the product is directly exposed to solar radiation; (b) indirect solar drying, commonly referred to as convective solar drying; and (c) mixed mode or hybrid solar drying^49–53^. Furthermore, solar dryers can be classified into two principal categories: natural convection solar dryers and forced convection solar dryers^53–57^. From a structural perspective, solar dryers can be subdivided into three major types: greenhouse types, collector types, and heat pump-assisted types^58,59^.

Many previous studies were performed on drying the marjoram leaves, but all of them were focused on physicochemical properties only, such as AlJuhaimi et al.^60^studied the effect of drying methods on the antioxidant capacity and bioactive and phenolic constituents in the aerial parts of marjoram grown naturally in the Taurus mountains in the Mediterranean region. Ganash^61^ investigated the potential of using dried marjoram leaves as a green inhibitor to prevent steel from rusting in an acidic solution. Raghavan et al.^62,63^ studied the effect of drying methods on the flavor quality of marjoram. And Szwedziak et al.^63^ studied the influence of biological preparations and drying methods on the content of essential oils in basil and marjoram.

To our knowledge, no publications in the literature describe the effects of drying methods, drying temperatures, and thin layer thickness on mathematical modeling, EMD, activation energy, drying parameters, and thermodynamic properties of ESML. So, the current study aimed to use an indirect solar dryer integrated with control units for temperature, humidity, and electrical heater (HSDS) for drying ESML and compare the obtained results with another oven drying (OD) under different drying temperatures and thin layer thicknesses.

## Materials and methods

### Experimental procedures

**Fig. 1:**
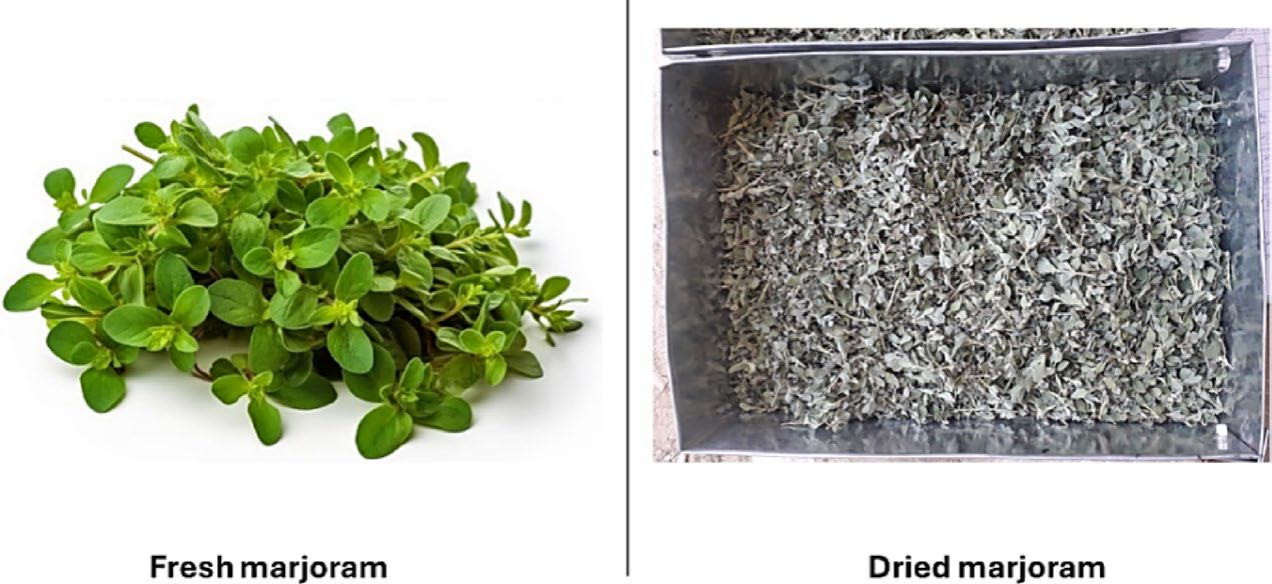
Fresh and dried Egyptian sweet marjoram leaves during the current study.

The fresh ESML *(Origanum majorana L.)* was collected from the Experimental Research Station, Faculty of Agriculture Farm, Moshtohor, Benha University. When initially harvested, the fresh ESML had an average MC of 82% (W.b.). Then, the fresh ESML was distributed at three-layer thicknesses of 1, 2, and 3 cm above the drying trays and put in the drying room. During the current study, two different drying systems were used for drying ESML: the first is a flat-plate solar dryer integrated with an electric heater (hybrid solar drying system (HSDS)), and the other is an oven drying (OD). Additionally, three drying system temperatures of 50, 60, and 70 °C and three-layer thicknesses of 1, 2, and 3 cm were established. To ensure uniform distribution and consistent drying conditions across all samples, drying molds with identical internal dimensions (30 cm in length, 20 cm in width, and 7 cm in height) were utilized. Based on the mold’s surface area and the bulk density of fresh marjoram leaves (0.15 g/cm³), 90 g of marjoram were evenly spread to achieve a layer thickness of approximately 1 cm. To prepare thicker layers, the sample weight was proportionally increased—180 g for a 2 cm layer and 270 g for a 3 cm layer—thereby maintaining uniform density and distribution across all tested thicknesses. The experimental design was split plot with three replicates. All tests on drying fresh ESML were done in the field at the Faculty of Agriculture Moshtohor, Benha University, Egypt, from summer 2022. These tests were done according to the rules set by International, National, and Benha University, which are in line with national and international laws and rules. During field tests, the average daily ambient air temperature, relative humidity, and solar radiation were 32.05 °C, 55.8%, and 640.05 W.h/m²/day, respectively. Where daily ambient air temperature, relative humidity, and solar radiation were recorded in Benha University, Egypt, from May to June 2022, and daily mean values ​​were then calculated based on the obtained data. Figure 1 shows fresh and dried ESML.

### Description of the HSDS

**Fig. 2:**
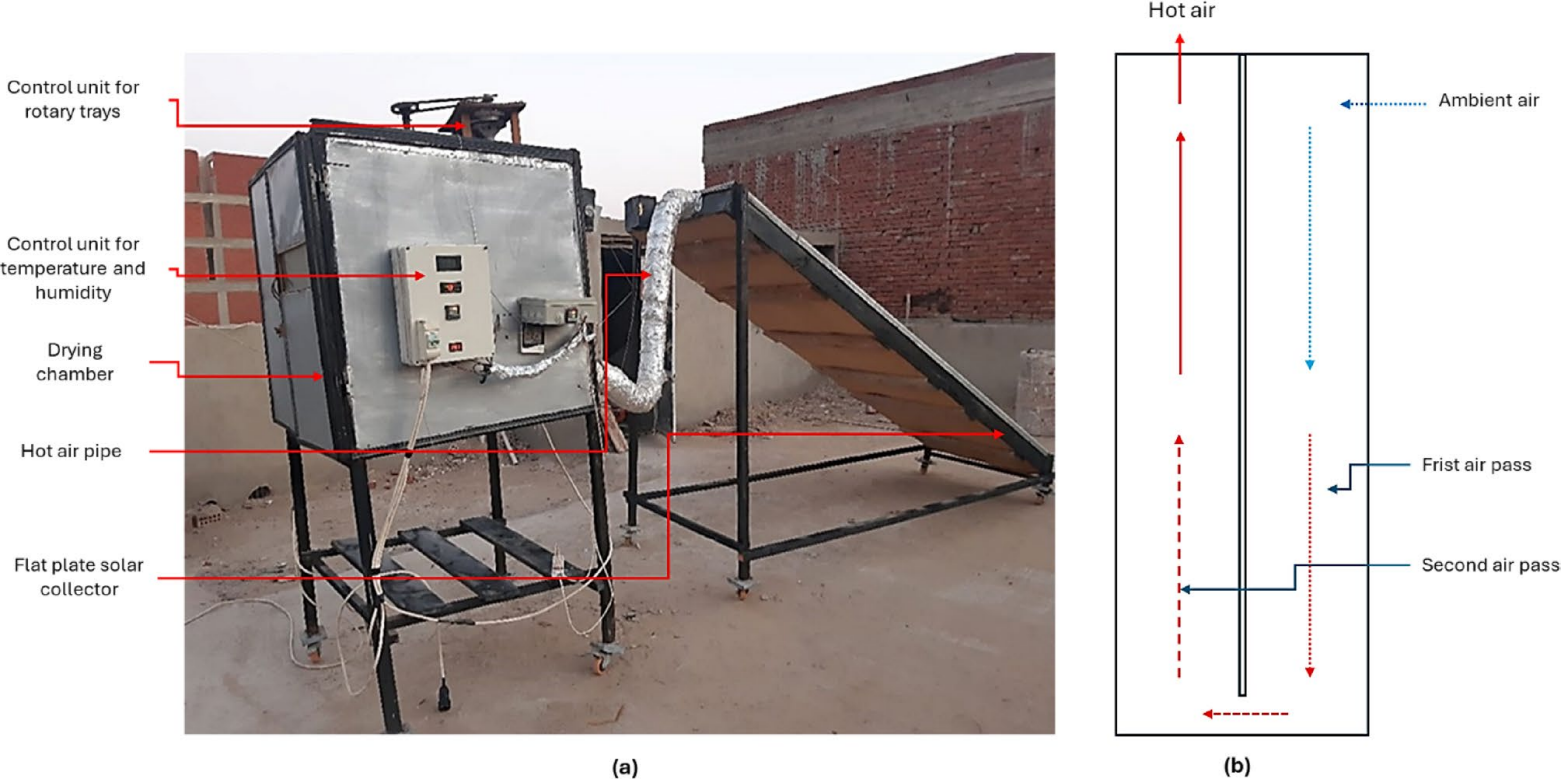
Main components of the hybrid solar drying system. Whereas (**a**) real photo of the HSDS, (**b**) double air circulation passes inside the flat plate solar collector.

**Fig. 3:**
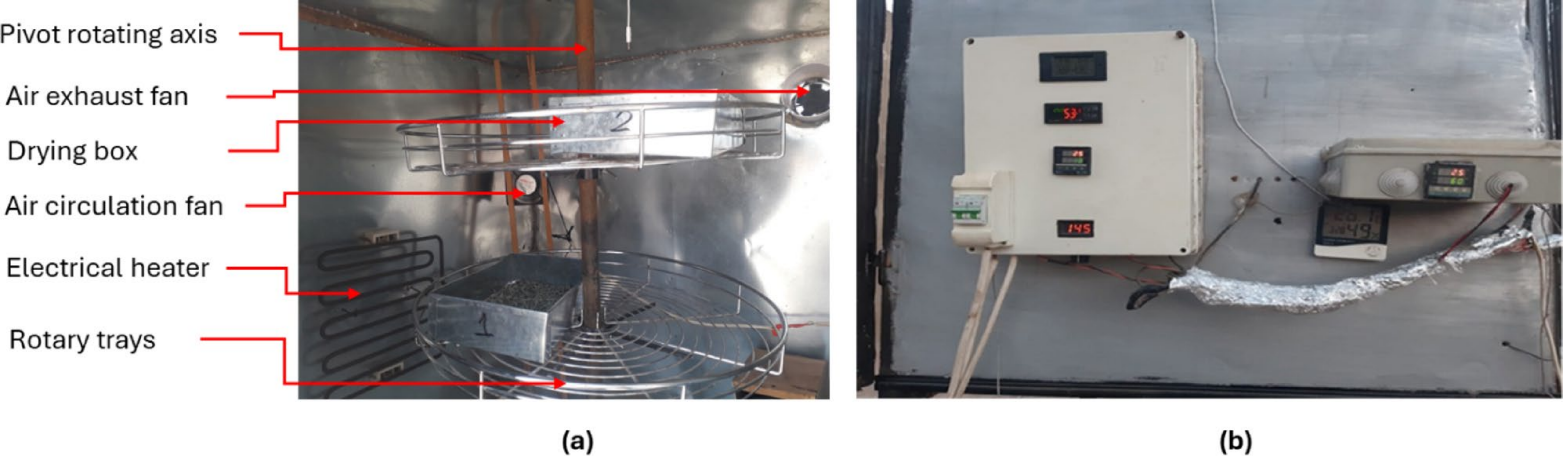
Internal components and control units of the hybrid solar drying system. Whereas (**a**). internal components of the drying room, and (**b**). control units for temperature, humidity and the electrical heater.

To achieve the objectives of the current study, a HSDS was designed, developed, and implemented. This system combines renewable solar energy with conventional electrical heating to provide an efficient and controllable drying environment. The HSDS is equipped with a Double Pass Flat Plate Solar Collector (DPFPSC), which functions as the primary solar heat source. Additionally, an auxiliary electric heater was integrated into the system to elevate and regulate the air temperature inside the drying chamber, particularly during periods of low solar radiation. The schematic layout of the developed HSDS is illustrated in Fig. 2a. The system comprises several key components:

I. Double Pass Flat Plate Solar Collector (DPFPSC) (Fig. 2b): The collector measures 4 m in length, 1 m in width, and 20 centimeters in depth. It is covered with a 3 mm thick transparent glass sheet to minimize heat loss while allowing maximum solar radiation transmission. The internal absorber plate is made of corrugated black-painted aluminum, selected for its high thermal conductivity and efficient heat absorption. The absorber is thermally insulated using a 5 cm thick layer of thermal wool to minimize heat dissipation.
II. Drying Chamber: The drying chamber, which serves as the core of the drying process, has cubic dimensions of 1.0 × 1.0 × 1.0 m. Both its inner and outer walls are constructed from 5 mm thick galvanized steel to ensure durability and resistance to corrosion. Thermal insulation is achieved through a composite of 2 cm thick thermal wool and 3 cm of foam, reducing heat losses and maintaining a stable internal temperature.
III. Drying Boxes (Fig. 3a): The drying chamber contains multiple drying boxes, each fabricated from stainless steel to ensure hygiene and corrosion resistance. These boxes measure 30 cm in length, 20 cm in width, and 7 cm in height. Their perforated bottoms allow for uniform airflow distribution through the drying material, thereby enhancing drying efficiency.
IV. Rotary Trays (Fig. 3a): To promote uniform drying and maximize contact between the hot air and the drying product, the chamber includes two rotary trays (shown in Fig. 3a). Each tray has a diameter of 60 cm and a height of 3 cm and is mounted on a centrally pivoted rotating axis, allowing for continuous motion and even heat exposure.
V. Electric Heater (Fig. 3a): A 2000 W electric heater is installed as a supplementary heat source. It assists in elevating the air temperature, particularly during periods of insufficient solar energy, and helps in reducing the relative humidity of the circulating air. This accelerates the drying process and significantly reduces the total drying time.
VI. Air Fans (Fig. 3a): Two high-performance air fans (Model C.C.P. Parm, Italy) are used in the system. These fans, each rated at 150 W with a capacity of 6.6 m³/h and a speed of 2800 rpm, are responsible for pushing heated air from the DPFPSC into the drying chamber and maintaining consistent air circulation within the chamber.
VII. Control system (Fig. 3b): In this study, two distinct control systems were employed to regulate the drying environment. The first system utilized a digital PID controller (Model: REX_C100), which was responsible for modulating the air temperature and relative humidity inside the drying chamber. This was achieved by controlling both the electrical heater and the circulation fan speed, ensuring precise environmental conditions for the drying process. The second control system managed the operation of the inlet and exhaust air fans, automatically adjusting their activation to help maintain the target temperature and humidity levels within the chamber. Together, these systems provided coordinated and stable control over the internal drying conditions, thereby enhancing drying efficiency and product quality.

This hybrid configuration allows for a reliable and energy-efficient drying operation, optimizing the use of renewable solar energy while ensuring performance consistency through electrical backup.

### Description of the oven drying (OD)

The drying chamber of the Hybrid Solar Drying System (HSDS) was repurposed as a conventional oven by utilizing only the electric heater, effectively isolating it from the solar component. This configuration was achieved by disengaging the intake fan connected to the solar collector and implementing a sealing gate mechanism to block the entry of hot air generated by solar radiation. As a result, heat was supplied exclusively through the electric heater, ensuring controlled and uniform temperature conditions within the drying chamber without any influence from solar input. The system setup during this mode of operation is illustrated in Fig. 3a.

### Evaluations of the HSDS

#### Drying characteristics

##### Moisture content (MC)

The initial MC of the fresh ESML was estimated under laboratory conditions according to AOAC^64^. Additionally, during the drying of ESML in the field using both drying systems until reaching the EMC, the interval weight was measured hourly, and the MC (w.b.) was calculated using Eq. (1), as mentioned by Elghazali et al.^65,66^ and Eke et al.^67^.

1 $$\:MC=\left[\frac{{W}_{w}-\:{W}_{d}}{{W}_{w}}\right]\times\:100$$

where, $\:{\text{W}}_{\text{w}}\:\text{a}\text{n}\text{d}\:{\text{W}}_{\text{d}}$ is the wet and dry weights of ESML samples.

##### Drying rate

The drying rate of different ESML samples were estimated using Eq. (2), as mentioned by Etim et al.^68^ and Ambawat et al.^68^ where Eq. (2), is the most widely used for estimating the drying rate because of its high accuracy.

2 $$\:Drying\:rate\:\:=\:\frac{{MC}_{(t+dt)}-{MC}_{t}}{{d}_{t}}$$

##### Moisture ratio (MR)

The moisture ratio of dried ESML samples at time (t) were estimated using Eq. (3)^69,54–57^,

3 $$\:MR=\frac{{M}_{t}-{M}_{e}}{{M}_{0}-{M}_{e}}\:$$

where, $\:{M}_{0}$ is the initial MC in %, $\:{M}_{e}$ is the EMC in %, and $\:{M}_{t}$ is the MC at any time in %.

#### Effective moisture diffusivity (EMD)

Most drying studies widely recognize diffusion as the primary process that transports moisture to the surface for evaporation. The EMD can be calculated from the slope of the normalized plot of the unachieved moisture ratio, ln MR over time, utilizing the following Equation^70,71^.

4 $$\:{ln}\left(MR\right)={ln}\left(\frac{8}{{\pi\:}^{2}}\right)-\:\left(\frac{{\pi\:}^{2}\times\:{D}_{eff}\times\:t}{4{L}^{2}}\right)$$

where, D_eff_ is the EMD in m^2^/s, and L is the half-thickness of n sample in m.

Finding the diffusion coefficient involves plotting experimental drying data in terms of ln (MR) versus time, s. The activation energy was also found using the law of Arrhenius, which is done in the same way as the diffusion coefficient^72–74^.

5 $$\:{D}_{eff}=\:{D}_{0}{exp}\left(-\frac{{E}_{a}}{RT}\right)$$

#### Mathematical modelling of the drying process

The drying data acquired were analyzed using nonlinear least squares regression to match eleven thin-layer drying models outlined in Table 1. Statistical analyses of the experimental data were conducted utilizing developed software produced by Öksüz and Buzrul^75^.

**Table 1:** Selected mathematical modeling to demonstrate the drying process of Egyptian sweet marjoram leaves.

No.	Model name	Model Equation*	Reference
1	Aghbashlo	$\:MR=\text{exp}\left(-\frac{{k}_{1}t}{1+{k}_{2}t}\right)$	75,76
2	Henderson - Pabis	$\:MR=a\:\text{e}\text{x}\text{p}\left(-kt\right)$	77–79
3	Lewis (Newton)	$\:\text{M}\text{R}=\text{e}\text{x}\text{p}\left(-\text{k}\text{t}\right)$	^77^
4	Logarithmic (Asymptotic)	$\:\text{M}\text{R}=\text{a}\text{*}\text{e}\text{x}\text{p}\left(-\text{k}\text{t}\right)+c$	77–79
5	Midilli	$\:\text{M}\text{R}=\text{a}\text{*}\text{e}\text{x}\text{p}\left(-\text{k}{\text{t}}^{n}\right)+bt$	77–79
6	Modified Midilli II	$\:\text{M}\text{R}=\text{a}\text{*}\text{e}\text{x}\text{p}\left(-\text{k}{\text{t}}^{n}\right)+b$	^80^
7	Modified Page	$\:\text{M}\text{R}=\text{e}\text{x}\text{p}\left(-{\left(\text{k}\text{t}\right)}^{\text{n}}\right)$	77–79
8	Page	$\:\text{M}\text{R}=\text{e}\text{x}\text{p}\left(-\text{k}{\text{t}}^{\text{n}}\right)$	^77–79^
9	Wang-Sigh	$\:MR=1+bt+a{t}^{2}$	77–79
10	Weibullian	$\:\text{M}\text{R}=\text{e}\text{x}\text{p}\left(-{\left(\frac{t}{\alpha\:}\right)}^{\beta\:}\right)$	80,81
11	Weibullian I	$\:\text{M}\text{R}={10}^{-{\left(\frac{t}{\delta\:}\right)}^{n}}$	80,81

* MR is the moisture ratio, dimensionless; k_1,_ k_2_ and k are the drying constants, h^− 1^; t is the drying time, h; a, b,c, n,ɤ, β and δ are the models constants, dimensionless.

The coefficient of determination (R^2^, the adjusted coefficient of determination ($\:{R}_{adj.}^{2}$) and root mean squared error (RMSE) are fundamental criteria for selecting the optimal model to characterize the drying curves. The optimal model was identified based on the criterion of minimal RMSE values and maximal R^2^ and $\:{R}_{adj.}^{2}$^82–86^. The following Eqs. (6–8) can be employed to calculate these parameters.

6 $$\:{R}^{2}=1-\frac{\sum\:_{i=1}^{N}{{(MR}_{pre,\:i}-{MR}_{obs,\:i})}^{2}}{\sum\:_{i=1}^{N}{{(\stackrel{-}{M}R}_{pre}-{MR}_{obs,\:i})}^{2}}$$

7 $$\:{R}_{adj.}^{2}=1-\left(1-{R}^{2}\right)*\frac{N-1}{N-n}$$

8 $$\:RMSE=\sqrt{\frac{1}{N}{\sum\:}_{i=1}^{N}{{(MR}_{pre,\:i}-{MR}_{obs,\:i})}^{2}}$$

where, MR_exp, i_ is experimental MR; MR_pre, i_ is predicted MR; N is number of observations; n is number of constants.

#### Thermodynamic properties

Drying is a critical unit operation in food, pharmaceutical, and agricultural industries, aimed at reducing the moisture content of materials to enhance shelf life, minimize microbial activity, and reduce weight for transport. While the kinetics of drying—such as moisture diffusivity and drying rate—have been extensively studied, understanding the thermodynamic properties associated with the drying process is equally vital. These properties provide insight into the energetic and molecular transformations that occur during moisture removal, enabling better design, control, and optimization of drying systems. The thermodynamic properties of particular interest in drying include enthalpy (ΔH), entropy (ΔS), and Gibbs free energy (ΔG). These parameters are typically derived from equilibrium moisture data and drying temperatures using the Clausius–Clapeyron equation and Gibbs–Helmholtz relation, and they help elucidate the nature of water binding and energy demands during the drying process. The thermodynamic properties related to the drying process of ESML were determined by following the methodology described by Jideani and Mpotokwana^87^ widely applied to several agricultural products (Eqs. 9–11).

The enthalpy change associated with the sorption process, also called the **isosteric heat of sorption**, represents the energy required to remove water molecules from the product surface. It is typically calculated using the Clausius–Clapeyron equation:

9 $$\:\varDelta\:H=\:{E}_{a}-RT\:\:\:$$

Entropy reflects the degree of disorder or randomness in the system. In drying, a **positive ΔS** implies that the transition from a bound water state to a vapor state increases system randomness, which is typical. The change in entropy is determined through:

10 $$\:\varDelta\:S=\:R\left(\text{ln}{D}_{o}-\text{ln}\frac{{K}_{B}}{{K}_{P}}-\:\text{ln}T\right)$$

Gibbs free energy indicates the spontaneity of the drying process. It is calculated using:

11 $$\:\varDelta\:G=\:\varDelta\:H-T\varDelta\:S\:\:$$

where ΔH is the enthalpy (J mol^− 1^), R is the universal gas constant (8.314 J mol^− 1^ K^− 1^), ΔS is the entropy (J mol^− 1^ K^− 1^), K_B_ is the Boltzmann’s constant (1.38 × 10^–23^ J K^− 1^), K_p_ is the Planck’s constant (6.626 × 10^− 34^ J s^− 1^), ΔG is the Gibbs free energy (J mol^− 1^), D_0_ is the pre-exponential factor.

##### Practical implications

Understanding these thermodynamic properties has substantial practical value.

- Energy Efficiency: Accurate enthalpy data help estimate the energy requirements of a drying system, allowing for the selection of optimal operating temperatures that minimize energy input.
- Drying Behavior: Entropy changes can indicate how easily water molecules transition to vapor. For example, higher entropy at elevated temperatures suggests more disorder and faster drying.
- Process Design and Control: Knowing whether a drying process is spontaneous (from ΔG) guides the selection of appropriate drying conditions or whether pre-treatments (e.g., salting, blanching) are needed to facilitate water removal.
- Material Stability: The thermodynamic parameters can also signal potential quality changes, as excessive heat (linked to high ΔH) can degrade sensitive compounds in herbs, fruits, or pharmaceuticals.

In summary, thermodynamic analysis complements kinetic studies by offering a molecular-level understanding of water removal during drying. This deeper insight leads to improved system design, energy savings, and enhanced product quality.

## Results and discussions

### Drying characteristics

**Fig. 4:**
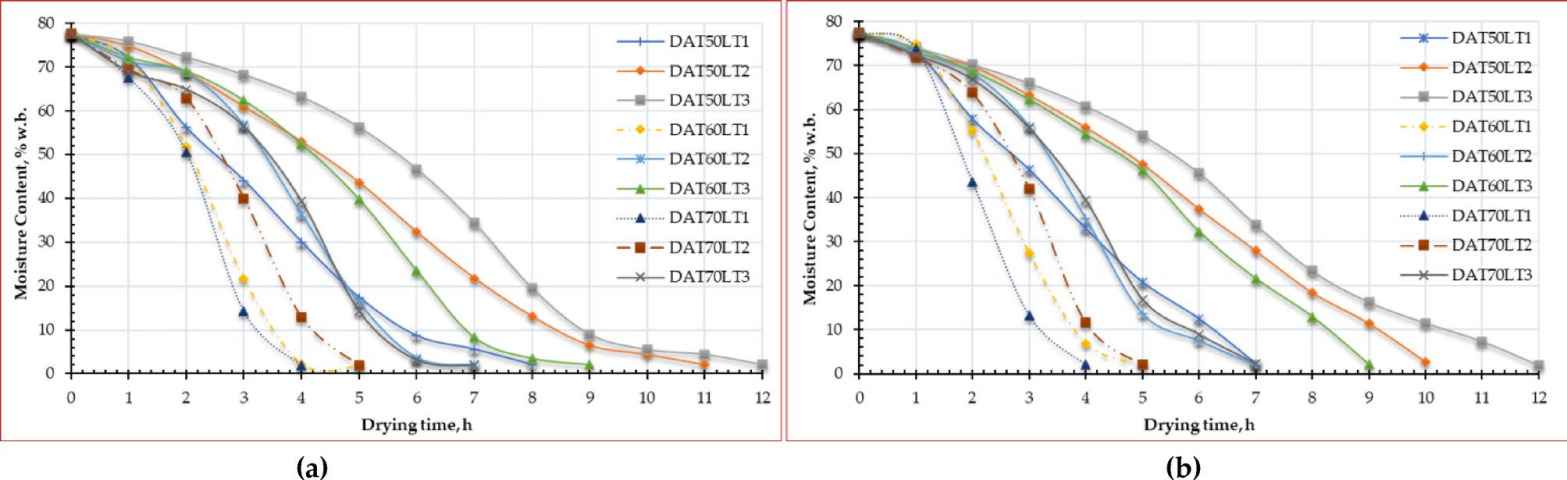
Moisture content of Egyptian sweet marjoram leaves at different drying air temperatures and layer thicknesses, (**a**) OD, and (**b**) HSDS.

Some agricultural products lose a lot of weight, which hurts their quality and makes them less profitable. For example, they might lose shape or texture, or the color might turn bad^88^. The primary cause of weight loss is the process of leaching and diffusion when water-soluble elements such as vitamins, tastes, minerals, carbohydrates, sugars, and proteins are released from plant tissue into the surroundings^89^. The initial MC in different ESML samples on a wet basis before the drying process was 77.50%, while Fig. 4 shows the MC in different ESML samples on a wet basis due to changes in drying temperature, and layer thickness. The illustrated data in the same figure showed that the final MC and drying time of ESML did not significantly differ for both drying method, while the drying time of ESML was decreased with increasing the air temperature, where the longest drying time of ESML was observed at an air temperature of 50 °C and layer thickness of 3 cm, while the shortest drying time of ESML was observed at an air temperature of 70 °C and layer thickness of 1 cm, meaning that the weight loss increases with increasing power. Similar observations were observed by many researchers including Kidmose and Kaack^90^ and Wang et al.^88^. Additionally, different ESML samples dried in OD achieved EMC ranging between 1.75% and 2.04% (w.b.). While different ESML samples dried in HSDS achieved EMC ranging between 1.92% and 2.67% (w.b.). Where in the initial stage of the drying process, the water molecules on the surface of the leaves gain enough energy from heat to overcome intermolecular forces and escape into the surrounding air as vapor. As the surface moisture dries up, water molecules from deeper within the leaf structure need to migrate to the surface for evaporation. Water molecules within the leaf structure move from areas of higher concentration (inside the leaf cells) to areas of lower concentration (the leaf surface) through a process called diffusion. This diffusion process is slower than the initial surface evaporation, contributing to the decreasing drying rate over time. As the MC decreases, water molecules become more tightly bound to the plant tissues. This increases the energy required to remove them, further slowing down the drying process.

**Fig. 5:**
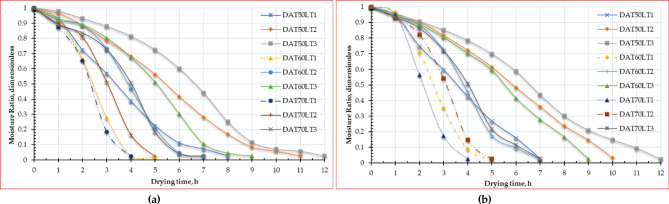
Moisture ratio of Egyptian sweet marjoram leaves at different drying temperatures and layers thicknesses, (**a**) OD, and (**b**) HSDS.

Figure 5 displays the relation between the moisture ratio and drying time curves of ESML at different drying air temperatures and layer thicknesses. The ESML was subjected to drying trials until they reached a state of EMC. Figure 5 demonstrates that raising the air temperature from 50 to 70 °C leads to a reduction in the time required for the finished product to dry by about 100%, 120%, and 71.4%, for layer thicknesses of 1 cm, 2 cm, and 3 cm, respectively, for OD, while the drying time was reduced by about 75%, 100%, and 71.4% for layer thicknesses of 1 cm, 2 cm, and 3 cm, respectively, for HSDS. This finding aligns with the outcomes reported by Beigi^91^ and Kaleta et al.^92^ where they found that, during the drying process, raising the air temperature from 50 to 60 °C resulted in enhanced mass transfer, decreased process duration, and lower energy usage^93^. Also, Kara and Doymaz’s^93^ reported that the increase in air temperature led to a decrease in the drying time. Additionally, Beigi^91^ observed that as the air temperature within the measured range rose, there was a corresponding rise in the quantity of moisture extracted from the product. Furthermore, we observed a drop in the MR curve as the hot air temperature increased, which aligns with the findings of Sharabiani et al.^94^. Where the inverse relationship between moisture ratio and drying time for ESML is a result of the decreasing moisture gradient within the ESML as drying progresses. This reduced gradient slows down the diffusion of water, leading to a longer drying time. Understanding this relationship is crucial for optimizing drying processes and preserving the quality of ESML.

**Fig. 6:**
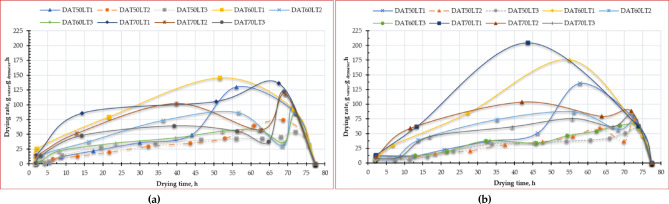
Drying rate of Egyptian sweet marjoram leaves at different drying temperatures and layers thicknesses, (**a**) OD, and (**b**) HSDS.

Figure 6 shows the DR of different ESML samples at different layer thicknesses, drying air temperatures and drying methods with respect to time. Where obtained results showed that the initial drying rates of different ESML samples dried in OD ranged between 31.63 and 136.45 kg _water_/kg _dry matter_/h. While the initial drying rates of different ESML samples dried in HSDS ranged between 46.34 and 88.3 kg _water_/kg _dry matter_/h. Where the maximum drying rate recorded was 136.45 kg _water_/kg _dry matter_/h at a 1 mm layer thickness for the OD-dried samples, whereas the highest drying rate for the HSDS-dried samples was 88.3 kg _water_/kg _dry matter_/h at a 1 mm layer thickness. The MC of a leafy crop has a direct and significant impact on its drying rate. As the MC decreases, the drying rate generally slows down. This relationship can be visualized in a typical drying curve, as shown in Fig. 6. Where ESML typically has high initial MCs, about 77.50% (w.b.). This high MC facilitates rapid evaporation initially. In the initial stage, the drying rate is relatively constant. This is because the MC is high, and the surface of the crop is saturated with water. The drying rate is primarily controlled by external factors like air temperature, humidity, and airflow. As the drying progresses, the MC decreases, and the drying rate slows down. This is because the moisture within the ESML needs to migrate to the surface for evaporation. This internal diffusion becomes the rate-limiting step. Similar results were obtained by many researchers, such as Nassef et al.^95^Sobukola et al.^96^ and Madan et al.^97^.

Table 2 lists the drying coefficient (k) and determination coefficient (R^2^ for ESML at different drying temperatures and layer thicknesses for OD and HSDS. The tabulated results showed that the drying coefficient (k) increases, as the drying air temperature within both drying systems rises. Because higher drying temperatures lead to a higher vapor pressure of water within the ESML. This increased vapor pressure creates a larger driving force for water molecules to evaporate from the surface. Also, increase the kinetic energy of water molecules within the ESML. This increased kinetic energy results in faster diffusion of water molecules from the interior to the surface, where they can evaporate. On the other hand, the obtained results showed that, the drying coefficient generally decreases with increasing layer thickness. As the layer thickness increases, the distance that water molecules must travel from the interior of the ESML to the surface for evaporation also increases. This longer diffusion path leads to increased resistance to water movement and a slower drying rate. Also, thicker layers of ESML have a smaller surface area to volume ratio compared to thinner layers. This means a smaller portion of the ESML is directly exposed to the drying environment, limiting the rate of moisture removal. Additionally, in thicker layers of ESML, temperature gradients can develop within the ESML during drying. The outer surface of ESML may be significantly warmer than the inner core, leading to uneven drying and potentially hindering moisture removal from the interior. These findings conformed well to the comparable pattern observed in the drying rate data. Doymaz^98^ Kaleta et al.^92^ and Meziane^99^ had similar observations. According to the coefficient of determination, the tabulated data showed that it has a direct relation with layer thickness, while it has an inverse relation with drying temperature.

**Table 2:** Drying coefficient and coefficient of determination of Egyptian sweet marjoram leaves at different drying temperatures and layers thickness for OD and HSDS.

Items	Drying system	DAT50			DAT60			DAT70		
		LT1	LT2	LT3	LT1	LT2	LT3	LT1	LT2	LT3
k	OD	-0.448	-0.325	-0.305	-0.867	-0.53	-0.41	-0.891	-0.688	-0.556
	HSD	-0.43	-0.275	-0.261	-0.743	-0.506	-0.321	-0.89	-0.682	-0.47
R^**2**^	OD	0.936	0.8959	0.8676	0.8706	0.843	0.8474	0.831	0.7896	0.836
	HSD	0.8295	0.7882	0.8308	0.8828	0.8549	0.7369	0.8604	0.7999	0.8378

### Effective moisture diffusivity (EMD)

**Fig. 7:**
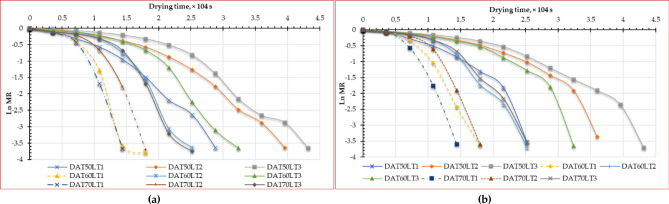
Ln MC vs. drying time of Egyptian sweet marjoram leaves at different drying temperatures and layers thicknesses, (**a**) OD, and (**b**) HSDS.

Figure 7 illustrates the EMD of ESML at various drying temperatures and layer thicknesses. The EMD is influenced by the reduced distance that moisture must traverse before to evaporate into the surrounding atmosphere. Moisture gradients generated within the meal during the drying process induce strains in the cellular structure^100^. This may result in structural failure, causing alterations to the material’s volume, shape, or dimensions. The duration of moisture diffusion from the interior to the exterior of food is influenced by the rupture of cell walls^101^. This aspect must be incorporated into mathematical models to ensure accurate predictions of sample MC during drying or to determine the suitable EMD^100^. A variety of elements, such as the pre-treatment solution, air temperatures, and the properties of the dried materials, influenced the EMD^53,102^. Figure 7 illustrates the correlation between the natural logarithm of moisture rate and drying time, considering drying temperatures and thin layer thickness. The coefficient of determination (R²) ranges from 0.7896 to 0.936 for OD and from 0.7369 to 0.8828 for HSDS, allowing for further calculation of the slope. Figure 7 clearly illustrates that the natural logarithm of the moisture rate has a downward tendency, particularly at greater thickness, where higher temperatures correspond to a more rapid decline. Comparable results have also been documented in other studies regarding the drying dynamics of agricultural goods^56,57,103^.

**Fig. 8:**
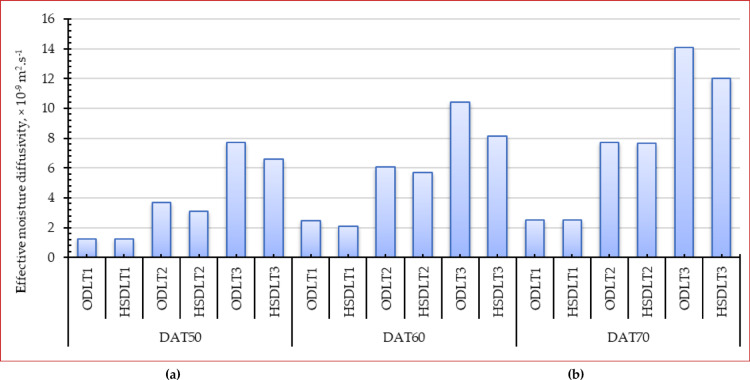
**E**ffective moisture diffusivity of Egyptian sweet marjoram leaves at different drying temperatures and layers thicknesses, (**a**) OD, and (**b**) HSDS.

Subsequently, EMD can be computed using Eq. (5) referenced above, and the results are depicted in Fig. 8. At a constant drying temperature, the EMD exhibited an upward trend as the thickness of the dry thin layer increased. Comparable detection was observed in expression at the same thin layer thickness, indicating that the water diffusion coefficient rises with an increase in temperature. At a drying temperature of 70 °C, the EMD for the 1.0, 2.0, and 3.0 cm layer thicknesses was 2.51 × 10^− 9^ m²·s⁻¹, 7.73 × 10^− 9^ m²·s⁻¹, and 14.1 × 10^− 9^ m²·s⁻¹, respectively, for the OD, and 2.50 × 10^− 9^ m²·s⁻¹, 7.67 × 10^− 9^ m²·s⁻¹, and 12 × 10^− 9^ m²·s⁻¹, respectively, for the HSDS. At a thickness of 1.0 cm, the maximum EMD was seen at a drying temperature of 70 °C, while the smallest EMD occurred at a drying temperature of 50 °C. Consequently, it can be inferred that augmenting the thickness and drying temperature of the thin layer enhances the internal pressure of the layer and elevates the activity of water molecules within the material, thereby facilitating moisture diffusion and subsequently improving the drying rate. This conclusion was corroborated by prior experiments on carrot slices^104^tea^105^ basil leaves^57^ and red sorghum^106^. Table 3 shows a comparison between the obtained EMD with previous studies.

**Table 3:** Comparison between the obtained EMD with previous studies.

Reference	Drying system	Dried product	EMD, m^2^/s
Ambawat et al.^103^	Fluidized Bed Dryer	Moringa leaves	3.59 to 2.92 × 10^− 10^
Seyedabadi^104^	Microwave	Basil leaves	1.624 to 7.652 × 10^− 10^
López-Ortiz et al.^105^	Solar greenhouses	Basil leaves	0.08 to 8.11 × 10^− 10^
Mbegbu et al.^106^	Vacuum oven dryer	Scent leaves	4.76 to 1.74 × 10^− 12^
Mbegbu et al.^106^	Vacuum oven dryer	Lemon basil leaves	4.80 to 2.06 × 10^− 12^
Altay et al.^107^	Microwave	Purple basil	0.162 to 7.09 × 10^− 8^
**Current study**	**HSDS**	**Marjoram leaves**	**14.1 × 10** ^**–9**^

### Activation energy

**Fig. 9:**
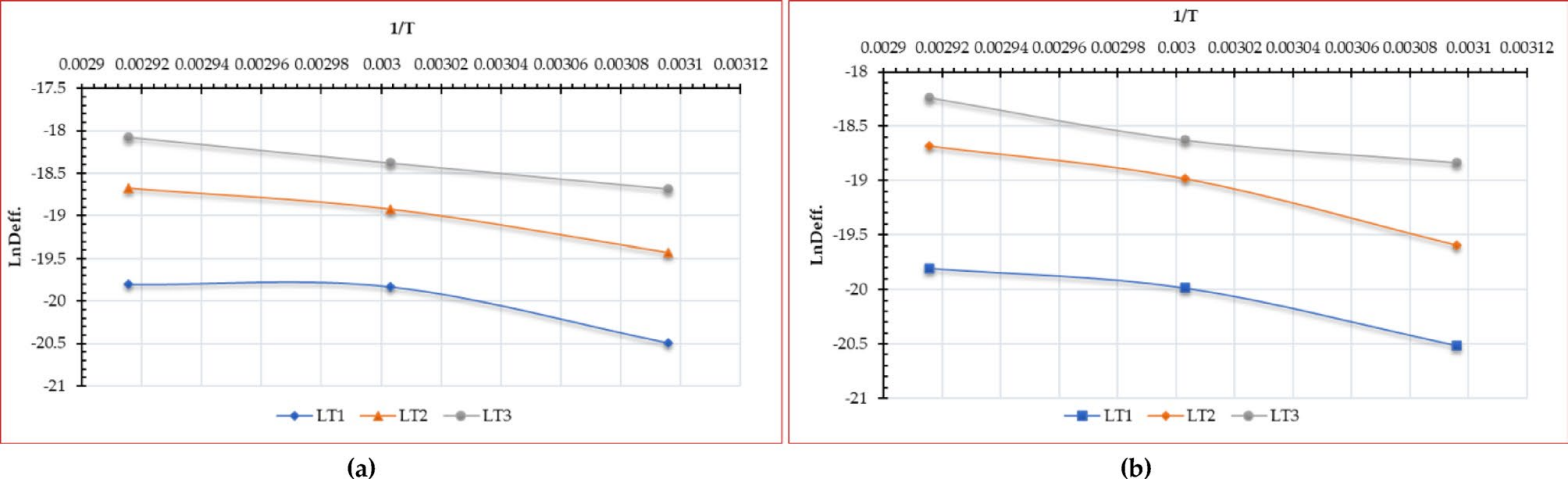
LnDeff. vs. 1/T of Egyptian sweet marjoram leaves at different drying temperatures and layers thicknesses, (**a**) OD, and (**b**) HSDS.

The activation energy can be computed using a method analogous to that employed for determining EMD. Utilized the EMD values collected previously to construct linear correlations with the inverse of the absolute drying temperature data, as illustrated in Fig. 9.

**Fig. 10:**
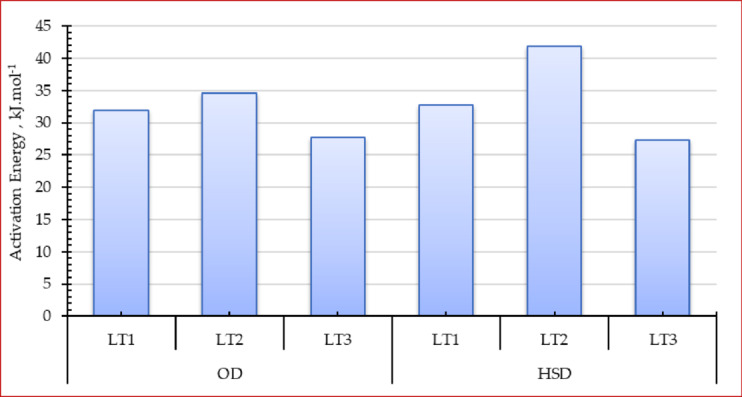
Activation energy of Egyptian sweet marjoram leaves at different drying systems and layers thicknesses.

Consequently, the activation energy is ascertained by calculating the slope of the linear correlations depicted in Fig. 10. Figure 10 illustrates that in this investigation, the activation energy values for thicknesses of 1.0, 2.0, and 3.0 cm were 31.95 kJ mol⁻¹, 34.64 kJ mol⁻¹, and 27.68 kJ mol⁻¹, respectively, for the OD, and 32.68 kJ mol⁻¹, 41.9 kJ mol⁻¹, and 27.35 kJ mol⁻¹, respectively, for the HSDS. The findings of this investigation are deemed plausible, supported by several pertinent literature sources indicating that the activation energy of vegetables and other plant-based materials ranges from 12.7 to 110 kJ mol⁻¹. Simultaneously, Luthra and Sadaka indicated that there is no substantial correlation between the augmentation of grain layer thickness and activation energy, which also elucidates the finding that the activation energy of moisture does not exhibit a definitive correlation with drying temperature and thickness observed in this study^107^. Moreover, these findings closely align with those reported by Shahi et al.^108^ who employed sun and vacuum dryers to dehydrate basil leaves at air temperatures of 45, 55, and 65 °C, indicating that the activation energy varied from 38.54 to 20.32 kJ mol⁻¹. Moreover, Martins et al.^109^ reported that the activation energy of basil leaves is 39.63 kJ mol⁻¹. Mbegbu et al.^110^ investigated the influence of varying air temperatures (30–70 °C) on the activation energy of lemon basil leaves utilizing a vacuum oven drier. They reported that the activation energy was 32.34 kJ·mol⁻¹.

### Mathematical modeling

Table 4 shows the mathematical model constants and statistical parameters of OD and HSDS for ESML under different drying air temperatures and layer thicknesses. Where eleven mathematical models were used to study the drying kinetics of ESML. In the beginning, the data of the MC of different dried samples was observed at different drying air temperatures and layer thicknesses. Then, the MC was converted into the moisture ratio expression using Eq. (3), and eleven mathematical models were used to compute curve fitting. Choosing an appropriate mathematical model is essential for precisely forecasting the drying characteristics of different items^111^. Nevertheless, the choice of the most suitable mathematical model for characterizing the drying characteristics of spices and herbs can’t exclusively rely on the quantity of constants. The selection procedure must be driven by statistical indications that have proven effective in choosing appropriate drying models, as documented in the literature.

**Fig. 11:**
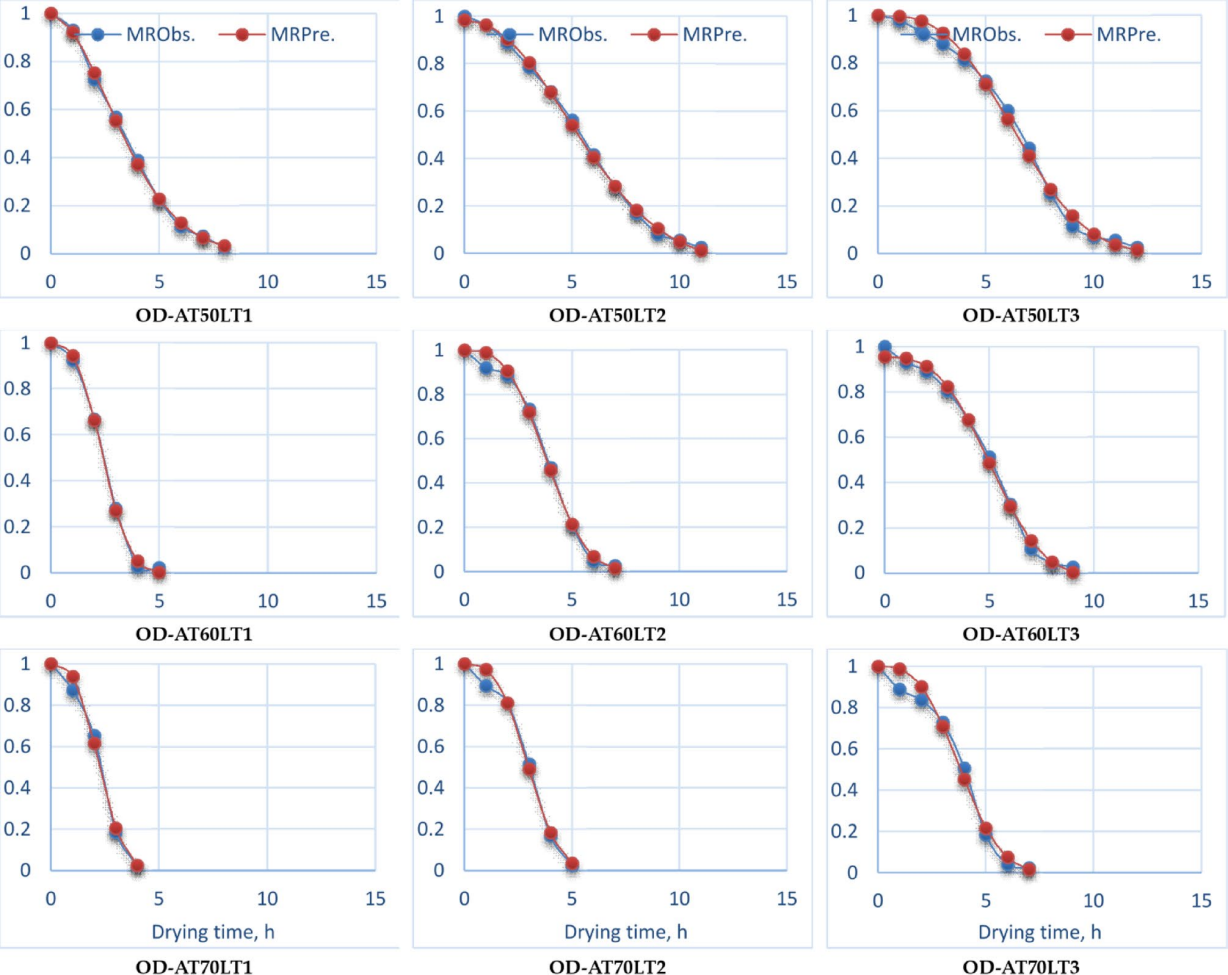
Observed and predicted MR for OD at the best mathematical model (Weibullian I).

**Fig. 12:**
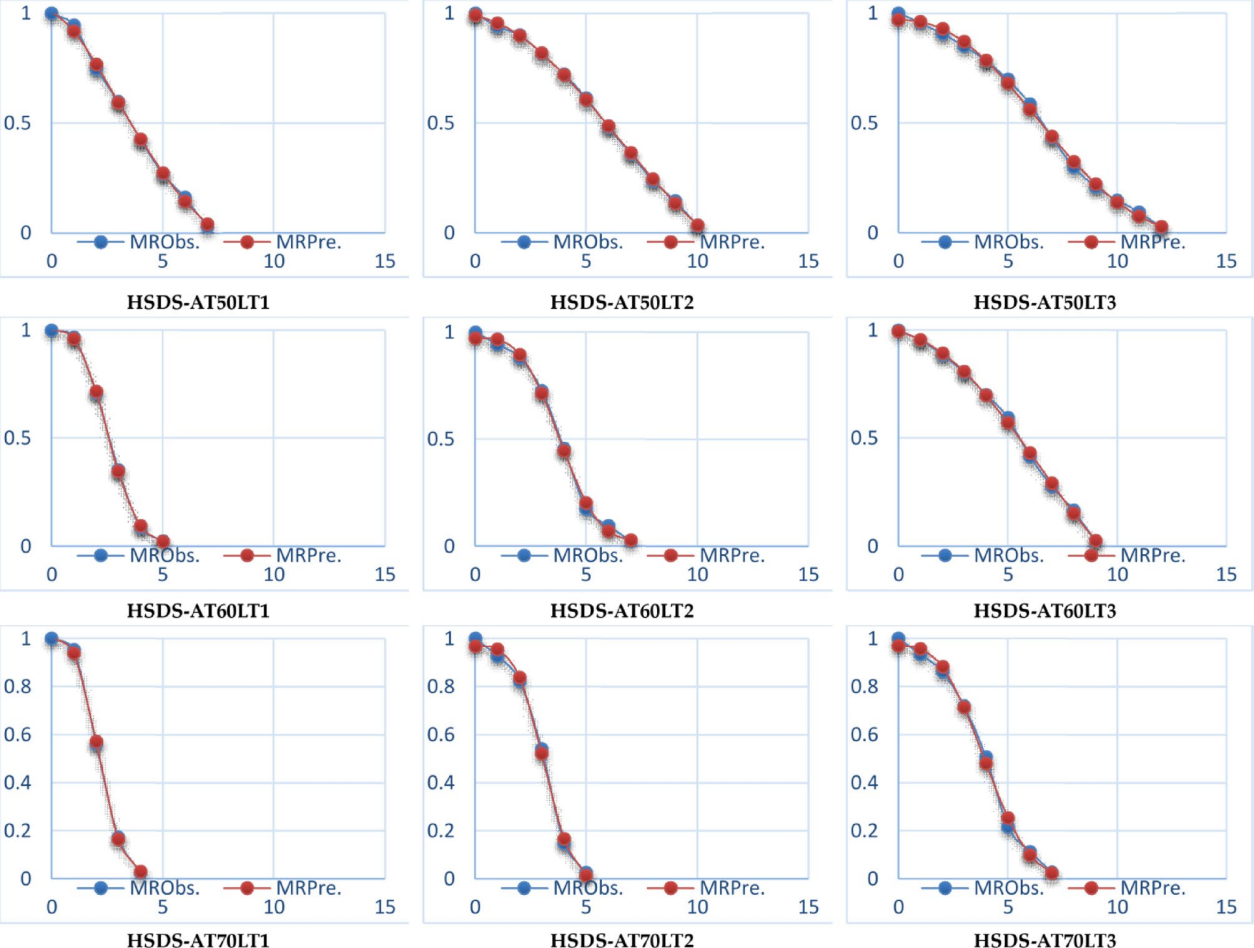
Observed and predicted MR for HSDS at the best mathematical model (Midilli I).

Hence, it’s crucial to meticulously evaluate these statistical indicators when choosing a mathematical model, guaranteeing that the mathematical model selection is based on well-informed decisions and supported by actual data. The findings of different statistical analysis, which are shown in Table 5, show that all drying kinetics had an overall high *R*^2^and $\:{R}_{adj.}^{2}$, and low *RMSE*, which are examples of statistical measures that were used to assess the quality of the fitted models. Many researchers demonstrated that the mathematical model most suited for defining the thin layer drying was the one with the greatest R^2^and $\:{R}_{adj.}^{2}$, and low *RMSE* values^82–84^. In addition, all eleven basic mathematical models were applied to predict the drying behavior of ESML during the drying process, where the Weibullian (I) drying model was the best models to represent the drying kinetics of ESML for OD, while the Midilli drying model was the best model to represent the drying kinetics of ESML for HSDS (Table 5; Figs. 11 and 12).

**Table 4:** Mathematical models’ constants values and goodness of fit indices results of OD and HSDS for ESML under different drying air temperature (DAT).

MMs	DAT, ºC	LT, cm	Parameters	Oven drying system (OD)							Parameters	Hybrid solar drying system (HSDS)						
				Models’ constants values				Goodness of fit indices				Models’ constants values				Goodness of fit indices		
				Values	S.E.	p-value	Sign. – Insign.	RMSE	R^2^	R^2^_adj_.		Values	S.E.	p-value	Sign.– Insign	RMSE	R^2^	R^2^_adj_.
**Aghbashlo**	**50**	**1**	**k**_**1**_	0.0400	0.0272	0.1846	Insign.	0.24622	0.61874	0.56427	**k**_**1**_	0.0400	0.0241	0.1478	**Insign.**	0.18817	0.76640	0.72747
			**k**_**2**_	-0.1250	0.0252	0.0016	Sign.				**k**_**2**_	-0.1429	0.0234	0.0009	Sign.			
		**2**	**k**_**1**_	0.0650	0.0042	2.39*10^− 8^	Sign.	0.02747	0.99494	0.99444	**k**_**1**_	0.0571	0.0024	1.80*10^− 9^	Sign.	0.01837	0.99735	0.99705
			**k**_**2**_	-0.0877	0.0036	2.85*10^− 10^	Sign.				**k**_**2**_	-0.0838	0.0021	1.64*10^− 11^	Sign.			
		**3**	**k**_**1**_	0.0469	0.0052	1.95E*10^− 6^	Sign.	0.04872	0.98528	0.98394	**k**_**1**_	0.0513	0.0039	4.70*10^− 8^	Sign.	0.03553	0.99073	0.98989
			**k**_**2**_	-0.0833	0.0046	1.63*10^− 9^	Sign.				**k**_**2**_	-0.0750	0.0034	1.82*10^− 10^	Sign.			
	**60**	**1**	**k**_**1**_	0.0400	0.0917	0.6850	InSign.	0.39275	0.35494	0.19367	**k**_**1**_	0.0400	0.0813	0.6486	InSign.	0.34858	0.47325	0.34156
			**k**_**2**_	-0.2000	0.1367	0.2173	InSign.				**k**_**2**_	-0.2000	0.1213	0.1746	InSign.			
		**2**	**k**_**1**_	0.0400	0.0224	0.1242	InSign.	0.17487	0.83806	0.81107	**k**_**1**_	0.0400	0.0233	0.1362	InSign.	0.18170	0.82415	0.79484
			**k**_**2**_	-0.1429	0.0217	0.0006	Sign.				**k**_**2**_	-0.1429	0.0226	0.0007	Sign.			
		**3**	**k**_**1**_	0.0604	0.0052	2.58*10^− 6^	Sign.	0.03246	0.99365	0.99286	**k**_**1**_	0.0564	0.0027	3.03*10^− 8^	Sign.	0.01993	0.99703	0.99666
			**k**_**2**_	-0.1111	0.0047	1.06*10^− 8^	Sign.				**k**_**2**_	-0.0961	0.0024	1.70*10^− 10^	Sign.			
	**70**	**1**	**k**_**1**_	0.0400	0.1762	0.8350	InSign.	0.44202	0.19152	-0.07798	**k**_**1**_	0.0400	0.1830	0.8410	InSign.	0.45903	0.18973	-0.08036
			**k**_**2**_	-0.2500	0.4039	0.5798	InSign.				**k**_**2**_	-0.2500	0.4194	0.5931	InSign.			
		**2**	**k**_**1**_	0.0400	0.0659	0.5765	InSign.	0.28232	0.60716	0.50894	**k**_**1**_	0.0400	0.0650	0.5717	InSign.	0.27867	0.63410	0.54263
			**k**_**2**_	-0.2000	0.0983	0.1115	InSign.				**k**_**2**_	-0.2000	0.0970	0.1082	InSign.			
		**3**	**k**_**1**_	0.0400	0.0230	0.1325	InSign.	0.17960	0.82398	0.79464	**k**_**1**_	0.0400	0.0202	0.0950	InSign.	0.15779	0.85794	0.83426
			**k**_**2**_	-0.1429	0.0223	0.0007	Sign.				**k**_**2**_	-0.1429	0.0196	0.0003	Sign.			
**Henderson - Pabis**	**50**	**1**	**k**	0.2820	0.0353	9.26*10^− 5^	Sign.	0.09552	0.94262	0.93442	**k**	0.2580	0.0372	0.0004	Sign.	0.10447	0.92799	0.91599
			**a**	1.1149	0.0802	2.36*10^− 6^	Sign.				**a**	1.1131	0.0872	1.41*10^− 5^	Sign.			
		**2**	**k**	0.1844	0.0241	1.71*10^− 5^	Sign.	0.11666	0.90879	0.89967	**k**	0.1584	0.0226	6.33*10^− 5^	Sign.	0.11800	0.89063	0.87847
			**a**	1.1546	0.0874	1.18*10^− 7^	Sign.				**a**	1.1330	0.0865	3.65*10^− 7^	Sign.			
		**3**	**k**	0.1569	0.0255	7.22*10^− 5^	Sign.	0.15462	0.85173	0.83825	**k**	0.1476	0.0198	1.30*10^− 5^	Sign.	0.12351	0.88799	0.87780
			**a**	1.1890	0.1107	3.60*10^− 7^	Sign.				**a**	1.1546	0.0872	4.21*10^− 8^	Sign.			
	**60**	**1**	**k**	0.4107	0.1129	0.0220	Sign.	0.18045	0.86383	0.82979	**k**	0.3725	0.1021	0.0218	Sign.	0.18174	0.85681	0.82102
			**a**	1.1262	0.1651	0.0024	Sign.				**a**	1.1385	0.1642	0.0023	Sign.			
		**2**	**k**	0.2554	0.0598	0.0053	Sign.	0.17500	0.83782	0.81079	**k**	0.2563	0.0577	0.0044	Sign.	0.16927	0.84738	0.82195
			**a**	1.1491	0.1457	0.0002	Sign.				**a**	1.1549	0.1410	0.0002	Sign.			
		**3**	**k**	0.2023	0.0384	0.0008	Sign.	0.15618	0.85304	0.83467	**k**	0.1701	0.0280	0.0003	Sign.	0.13032	0.87317	0.85732
			**a**	1.1543	0.1217	1.26*10^− 5^	Sign.				**a**	1.1370	0.0982	2.81*10^− 6^	Sign.			
	**70**	**1**	**k**	0.4175	0.1361	0.0547	**InSign.**	0.19337	0.84527	0.79369	**k**	0.4351	0.1441	0.0568	**InSign.**	0.20079	0.84497	0.79329
			**a**	1.1009	0.1793	0.0087	Sign.				**a**	1.1210	0.1869	0.0093	Sign.			
		**2**	**k**	0.3160	0.0917	0.0262	Sign.	0.18590	0.82966	0.78708	**k**	0.3126	0.0963	0.0315	Sign.	0.19943	0.81260	0.76575
			**a**	1.1190	0.1644	0.0024	Sign.				**a**	1.1331	0.1761	0.0030	Sign.			
		**3**	**k**	0.2567	0.0603	0.0053	Sign.	0.17279	0.83708	0.80993	**k**	0.2453	0.0527	0.0035	Sign.	0.15907	0.85562	0.83156
			**a**	1.1307	0.1440	0.0002	Sign.				**a**	1.1438	0.1316	0.0001	Sign.			
**Lewis (Newton)**	**50**	**1**	**k**	0.2538	0.0293	2.43*10^− 5^	Sign.	0.10266	0.92426	0.92426	**k**	0.2297	0.0297	0.0001	Sign.	0.11053	0.90597	0.90597
		**2**	**k**	0.1589	0.0195	5.45*10^− 6^	Sign.	0.12936	0.87663	0.87663	**k**	0.1361	0.0173	1.38*10^− 5^	Sign.	0.12727	0.85863	0.85863
		**3**	**k**	0.1304	0.0200	2.90*10^− 5^	Sign.	0.16919	0.80632	0.80632	**k**	0.1254	0.0155	3.38*10^− 6^	Sign.	0.13602	0.85179	0.85179
	**60**	**1**	**k**	0.3690	0.0888	0.0089	Sign.	0.17388	0.84197	0.84197	**k**	0.3286	0.0800	0.0093	Sign.	0.17780	0.82870	0.82870
		**2**	**k**	0.2209	0.0459	0.0019	Sign.	0.17723	0.80594	0.80594	**k**	0.2204	0.0448	0.0017	Sign.	0.17354	0.81286	0.81286
		**3**	**k**	0.1736	0.0295	0.0002	Sign.	0.16319	0.81951	0.81951	**k**	0.1452	0.0212	7.60*10^− 5^	Sign.	0.13856	0.83871	0.83871
	**70**	**1**	**k**	0.3793	0.1014	0.0201	Sign.	0.17682	0.82751	0.82751	**k**	0.3897	0.1101	0.0240	Sign.	0.18666	0.82136	0.82136
		**2**	**k**	0.2796	0.0682	0.0094	Sign.	0.17790	0.80502	0.80502	**k**	0.2727	0.0720	0.0128	Sign.	0.19200	0.78288	0.78288
		**3**	**k**	0.2259	0.0455	0.0016	Sign.	0.17185	0.81198	0.81198	**k**	0.2119	0.0405	0.0012	Sign.	0.16286	0.82344	0.82344
**Logarithmic (Asymptotic)**	**50**	**1**	**k**	0.1110	0.0467	0.0551	InSign.	0.05405	0.98425	0.97901	**k**	0.0108	0.0376	0.7851	InSign.	0.03576	0.99297	0.99016
			**a**	1.8380	0.4884	0.0094	Sign.				**a**	14.0632	47.0570	0.7771	InSign.			
			**c**	-0.7787	0.5121	0.1792	InSign.				**c**	-13.0223	47.0761	0.7931	InSign.			
		**2**	**k**	0.0187	0.0296	0.5437	InSign.	0.05467	0.98197	0.97797	**k**	0.0008	0.0309	0.9790	InSign.	0.04581	0.98535	0.98169
			**a**	5.9971	8.5636	0.5014	InSign.				**a**	121.2596	4,444.8786	0.9789	InSign.			
			**c**	-4.9300	8.5904	0.5801	InSign.				**c**	-120.1835	4,444.9021	0.9791	InSign.			
		**3**	**k**	0.0008	0.0398	0.9839	InSign.	0.08601	0.95829	0.94995	**k**	0.0011	0.0270	0.9690	InSign.	0.05435	0.98028	0.97634
			**a**	117.9823	5,677.6642	0.9838	InSign.				**a**	83.8750	2,091.3445	0.9688	InSign.			
			**c**	-116.8720	5,677.7064	0.9840	InSign.				**c**	-82.7963	2,091.3711	0.9692	InSign.			
	**60**	**1**	**k**	0.0463	0.1842	0.8177	InSign.	0.12788	0.94871	0.91452	**k**	0.0025	0.1707	0.9892	InSign.	0.11702	0.95547	0.92579
			**a**	5.5071	19.4838	0.7958	InSign.				**a**	90.4707	6,146.8193	0.9892	InSign.			
			**c**	-4.4336	19.5500	0.8352	InSign.				**c**	-89.3844	6,146.8830	0.9893	InSign.			
		**2**	**k**	0.0015	0.0991	0.9884	InSign.	0.10299	0.95319	0.93447	**k**	0.0009	0.0971	0.9928	InSign.	0.10063	0.95505	0.93707
			**a**	106.3304	6,915.8089	0.9883	InSign.				**a**	174.7632	18,419.9967	0.9928	InSign.			
			**c**	-105.2326	6,915.8644	0.9884	InSign.				**c**	-173.6649	18,420.0510	0.9928	InSign.			
		**3**	**k**	0.0009	0.0581	0.9885	InSign.	0.08315	0.96356	0.95314	**k**	0.0007	0.0454	0.9875	InSign.	0.05870	0.97748	0.97105
			**a**	143.3654	9,519.8106	0.9884	InSign.				**a**	152.8733	9,359.3937	0.9874	InSign.			
			**c**	-142.2752	9,519.8540	0.9885	InSign.				**c**	-151.7834	9,359.4244	0.9875	InSign.			
	**70**	**1**	**k**	0.0013	0.2493	0.9965	InSign.	0.12293	0.95831	0.91663	**k**	0.0011	0.2687	0.9971	InSign.	0.13697	0.95190	0.90381
			**a**	211.1256	41,952.2453	0.9964	InSign.				**a**	246.0130	59,422.7599	0.9971	InSign.			
			**c**	-210.0513	41,952.3109	0.9965	InSign.				**c**	-244.9237	59,422.8330	0.9971	InSign.			
		**2**	**k**	0.0011	0.1760	0.9953	InSign.	0.11298	0.95281	0.92136	**k**	0.0012	0.1975	0.9955	InSign.	0.12897	0.94122	0.90204
			**a**	188.8084	29,677.1669	0.9953	InSign.				**a**	178.5587	29,284.7132	0.9955	InSign.			
			**c**	-187.7140	29,677.2285	0.9954	InSign.				**c**	-177.4447	29,284.7835	0.9955	InSign.			
		**3**	**k**	0.0010	0.1008	0.9924	InSign.	0.10311	0.95165	0.93231	**k**	0.0010	0.0863	0.9909	InSign.	0.08692	0.96408	0.94971
			**a**	157.3968	15,713.6646	0.9924	InSign.				**a**	150.5448	12,505.4648	0.9909	InSign.			
			**c**	-156.3181	15,713.7202	0.9924	InSign.				**c**	-149.4519	12,505.5117	0.9909	InSign.			
**Midilli**	**50**	**1**	**k**	0.0843	0.0118	0.0008	Sign.	0.01774	0.99859	0.99774	**k**	0.0726	0.0117	0.0034	Sign.	0.01976	0.99828	0.99699
			**a**	1.0011	0.0161	2.02*10^− 8^	Sign.				**a**	1.0108	0.0185	6.75*10^− 7^	Sign.			
			**b**	-0.0017	0.0031	0.6062	InSign.				**b**	-0.0208	0.0109	0.1298	InSign.			
			**n**	1.7596	0.1078	1.57*10^− 5^	Sign.				**n**	1.6197	0.1482	0.0004	Sign.			
		**2**	**k**	0.0179	0.0034	0.0007	Sign.	0.01701	0.99845	0.99787	**k**	0.0116	0.0014	8.13*10^− 5^	Sign.	0.00971	0.99942	0.99918
			**a**	0.9836	0.0127	8.55*10^− 13^	Sign.				**a**	0.9896	0.0079	5.39*10^− 13^	Sign.			
			**b**	-0.0028	0.0022	0.2325	InSign.				**b**	-0.0221	0.0045	0.0018	Sign.			
			**n**	2.1517	0.1116	5.43*10^− 8^	Sign.				**n**	2.0656	0.0696	1.27*10^− 8^	Sign.			
		**3**	**k**	0.0017	0.0008	0.0530	InSign.	0.02782	0.99607	0.99477	**k**	0.0073	0.0020	0.0047	Sign.	0.02092	0.99737	0.99649
			**a**	0.9630	0.0164	6.23*10^− 13^	Sign.				**a**	0.9720	0.0144	1.72*10^− 13^	Sign.			
			**b**	0.0008	0.0020	0.6870	InSign.				**b**	-0.0031	0.0030	0.3242	InSign.			
			**n**	3.1985	0.2317	2.31*10^− 7^	Sign.				**n**	2.3750	0.1475	6.10*10^− 8^	Sign.			
	**60**	**1**	**k**	0.0510	0.0140	0.0677	InSign.	0.02447	0.99875	0.99687	**k**	0.0477	0.0054	0.0124	Sign.	**0.01020**	**0.99977**	**0.99944**
			**a**	0.9875	0.0209	0.0004	Sign.				**a**	1.0033	0.0087	7.54*10^− 5^	Sign.			
			**b**	0.0011	0.0047	0.8379	InSign.				**b**	0.0029	0.0023	0.3254	InSign.			
			**n**	2.9568	0.2691	0.0082	Sign.				**n**	2.8498	0.1068	0.0014	Sign.			
		**2**	**k**	0.0073	0.0033	0.0901	InSign.	0.02854	0.99712	0.99497	**k**	0.0097	0.0040	0.0728	InSign.	0.02804	0.99721	0.99512
			**a**	0.9611	0.0203	1.19*10^− 6^	Sign.				**a**	0.9723	0.0203	1.14*10^− 6^	Sign.			
			**b**	0.0004	0.0043	0.9332	InSign.				**b**	0.0033	0.0041	0.4619	InSign.			
			**n**	3.3266	0.3125	0.0004	Sign.				**n**	3.1938	0.2919	0.0004	Sign.			
		**3**	**k**	0.0052	0.0025	0.0813	InSign.	0.03143	0.99554	0.99330	**k**	0.0084	0.0020	0.0051	Sign.	0.01436	0.99885	0.99827
			**a**	0.9553	0.0213	8.23*10^− 9^	Sign.				**a**	0.9945	0.0119	2.00*10^− 10^	Sign.			
			**b**	-0.0019	0.0041	0.6601	InSign.				**b**	-0.0303	0.0083	0.0106	Sign.			
			**n**	3.0120	0.2883	4.51*10^− 5^	Sign.				**n**	2.2561	0.1054	6.77*10^− 7^	Sign.			
	**70**	**1**	**k**	0.0464	0.0380	0.4371	InSign.	0.06733	0.99375	0.97499	**k**	0.0775	0.0163	0.1319	InSign.	0.02078	0.99945	0.99779
			**a**	0.9658	0.0580	0.0382	Sign.				**a**	1.0090	0.0187	0.0118	Sign.			
			**b**	-0.0020	0.0226	0.9435	InSign.				**b**	0.0050	0.0063	0.5729	InSign.			
			**n**	3.1867	0.8822	0.1719	InSign.				**n**	2.9152	0.2427	0.0529	InSign.			
		**2**	**k**	0.0176	0.0113	0.2600	InSign.	0.04452	0.99511	0.98779	**k**	0.0116	0.0071	0.2426	InSign.	0.03811	0.99658	0.99145
			**a**	0.9627	0.0359	0.0014	Sign.				**a**	0.9682	0.0294	0.0009	Sign.			
			**b**	-0.0057	0.0145	0.7325	InSign.				**b**	-0.0015	0.0101	0.8971	InSign.			
			**n**	3.2286	0.5586	0.0287	Sign.				**n**	3.6027	0.5168	0.0200	Sign.			
		**3**	**k**	0.0050	0.0044	0.3172	InSign.	0.05023	0.99082	0.98394	**k**	0.0127	0.0056	0.0854	InSign.	0.03194	0.99612	0.99321
			**a**	0.9328	0.0348	1.15*10^− 5^	Sign.				**a**	0.9705	0.0241	2.27*10^− 6^	Sign.			
			**b**	-0.0011	0.0074	0.8876	InSign.				**b**	-0.0005	0.0063	0.9393	InSign.			
			**n**	3.5379	0.5998	0.0041	Sign.				**n**	2.8905	0.3125	0.0008	Sign.			
**Modified Midilli I I**	**50**	**1**	**k**	0.0848	0.0119	0.0009	Sign.	0.01768	0.99860	0.99775	**k**	0.0737	0.0093	0.0014	Sign.	0.01926	0.99837	0.99715
			**a**	1.0182	0.0380	1.36*10^− 6^	Sign.				**a**	1.2636	0.1759	0.0020	Sign.			
			**b**	-0.0170	0.0302	0.5980	InSign.				**b**	-0.2537	0.1684	0.2064	InSign.			
			**n**	1.7477	0.1182	2.56*10^− 5^	Sign.				**n**	1.5357	0.1591	0.0006	Sign.			
		**2**	**k**	0.0186	0.0038	0.0012	Sign.	0.01720	0.99841	0.99782	**k**	0.0169	0.0018	3.13*10^− 5^	Sign.	0.01230	0.99908	0.99868
			**a**	1.0180	0.0366	3.00*10^− 9^	Sign.				**a**	1.6760	0.3274	0.0014	Sign.			
			**b**	-0.0353	0.0303	0.2784	InSign.				**b**	-0.6870	0.3224	0.0706	InSign.			
			**n**	2.1254	0.1270	1.65*10^− 7^	Sign.				**n**	1.6999	0.1232	2.49*10^− 6^	Sign.			
		**3**	**k**	0.0015	0.0008	0.0764	InSign.	0.02745	0.99618	0.99490	**k**	0.0076	0.0024	0.0109	Sign.	0.02145	0.99724	0.99631
			**a**	0.9459	0.0315	2.44*10^− 10^	Sign.				**a**	1.0065	0.0543	1.78*10^− 8^	Sign.			
			**b**	0.0166	0.0243	0.5129	InSign.				**b**	-0.0372	0.0477	0.4553	InSign.			
			**n**	3.2648	0.2627	5.70*10^− 7^	Sign.				**n**	2.3588	0.1822	4.02*10^− 7^	Sign.			
	**60**	**1**	**k**	0.0505	0.0145	0.0731	InSign.	0.02445	0.99875	0.99687	**k**	0.0467	0.0059	0.0156	Sign.	0.01067	0.99975	0.99938
			**a**	0.9820	0.0338	0.0012	Sign.				**a**	0.9893	0.0168	0.0003	Sign.			
			**b**	0.0056	0.0238	0.8356	InSign.				**b**	0.0147	0.0126	0.3625	InSign.			
			**n**	2.9679	0.2911	0.0095	Sign.				**n**	2.8704	0.1251	0.0019	Sign.			
		**2**	**k**	0.0069	0.0034	0.1117	InSign.	0.02832	0.99717	0.99505	**k**	0.0085	0.0038	0.0913	InSign.	0.02700	0.99741	0.99547
			**a**	0.9521	0.0394	1.74*10^− 5^	Sign.				**a**	0.9426	0.0361	1.28*10^− 5^	Sign.			
			**b**	0.0084	0.0308	0.7994	InSign.				**b**	0.0301	0.0276	0.3360	InSign.			
			**n**	3.3721	0.3512	0.0007	Sign.				**n**	3.2934	0.3271	0.0005	Sign.			
		**3**	**k**	0.0051	0.0027	0.1118	InSign.	0.03184	0.99542	0.99313	**k**	0.0146	0.0024	0.0008	Sign.	0.01673	0.99843	0.99765
			**a**	0.9636	0.0502	1.29*10^− 6^	Sign.				**a**	1.8004	0.5714	0.0198	Sign.			
			**b**	-0.0105	0.0409	0.8051	InSign.				**b**	-0.8120	0.5647	0.2005	InSign.			
			**n**	3.0275	0.3342	0.0001	Sign.				**n**	1.8007	0.1872	7.23*10^− 5^	Sign.			
	**70**	**1**	**k**	0.0443	0.0393	0.4622	InSign.	0.06754	0.99371	0.97483	**k**	0.0759	0.0181	0.1488	InSign.	0.02191	0.99938	0.99754
			**a**	0.9596	0.1191	0.0786	InSign.				**a**	0.9902	0.0375	0.0241	Sign.			
			**b**	0.0038	0.0954	0.9746	InSign.				**b**	0.0197	0.0286	0.6163	InSign.			
			**n**	3.2638	1.0018	0.1896	InSign.				**n**	2.9407	0.2897	0.0625	InSign.			
		**2**	**k**	0.0173	0.0130	0.3140	InSign.	0.04596	0.99480	0.98699	**k**	0.0110	0.0075	0.2821	InSign.	0.03828	0.99655	0.99137
			**a**	0.9731	0.0986	0.0101	Sign.				**a**	0.9625	0.0658	0.0046	Sign.			
			**b**	-0.0143	0.0839	0.8801	InSign.				**b**	0.0037	0.0544	0.9523	InSign.			
			**n**	3.2743	0.6861	0.0412	Sign.				**n**	3.6704	0.6030	0.0259	Sign.			
		**3**	**k**	0.0046	0.0044	0.3604	InSign.	0.05035	0.99078	0.98386	**k**	0.0120	0.0060	0.1166	InSign.	0.03192	0.99612	0.99322
			**a**	0.9270	0.0671	0.0002	Sign.				**a**	0.9633	0.0586	8.03*10^− 5^	Sign.			
			**b**	0.0034	0.0525	0.9518	InSign.				**b**	0.0055	0.0484	0.9151	InSign.			
			**n**	3.6108	0.6806	0.0061	Sign.				**n**	2.9394	0.3695	0.0014	Sign.			
**Modified Page**	**50**	**1**	**k**	0.2487	0.0030	9.16*10^− 12^	Sign.	0.01550	0.99849	0.99827	**k**	0.2356	0.0048	4.51*10^− 9^	Sign.	0.02690	0.99523	0.99443
			**n**	1.7959	0.0566	7.99*10^− 9^	Sign.				**n**	1.8631	0.1061	2.19*10^− 6^	Sign.			
		**2**	**k**	0.1606	0.0018	7.96*10^− 16^	Sign.	0.01985	0.99736	0.99710	**k**	0.1482	0.0025	6.40*10^− 13^	Sign.	0.03080	0.99255	0.99172
			**n**	2.1500	0.0749	6.10*10^− 11^	Sign.				**n**	2.1943	0.1235	2.56*10^− 8^	Sign.			
		**3**	**k**	0.1373	0.0019	4.72*10^− 16^	Sign.	0.03081	0.99411	0.99358	**k**	0.1328	0.0018	2.59*10^− 16^	Sign.	0.02623	0.99495	0.99449
			**n**	2.8814	0.1586	1.50*10^− 9^	Sign.				**n**	2.3025	0.1011	1.32*10^− 10^	Sign.			
	**60**	**1**	**k**	0.3663	0.0051	2.25*10^− 7^	Sign.	0.01881	0.99852	0.99815	**k**	0.3391	0.0025	1.80*10^− 8^	Sign.	0.01034	0.99954	0.99942
			**n**	2.8592	0.1559	5.21*10^− 5^	Sign.				**n**	2.8226	0.0810	4.05*10^− 6^	Sign.			
		**2**	**k**	0.2309	0.0043	2.63*10^− 9^	Sign.	0.03219	0.99451	0.99360	**k**	0.2324	0.0037	1.18*10^− 9^	Sign.	0.02756	0.99596	0.99528
			**n**	3.0150	0.2283	1.17*10^− 9^	Sign.				**n**	2.9084	0.1866	4.42*10^− 6^	Sign.			
		**3**	**k**	0.1804	0.0036	2.89*10^− 11^	Sign.	0.03771	0.99143	0.99036	**k**	0.1604	0.0033	3.46*10^− 11^	Sign.	0.03690	0.98983	0.98856
			**n**	2.7340	0.2074	1.05*10^− 6^	Sign.				**n**	2.3633	0.1687	6.52*10^− 7^	Sign.			
	**70**	**1**	**k**	0.3900	0.0134	8.91*10^− 5^	Sign.	0.04548	0.99144	0.98859	**k**	0.4073	0.0054	5.17*10^− 6^	Sign.	0.01713	0.99887	0.99850
			**n**	2.9214	0.4004	0.0053	Sign.				**n**	2.8987	0.1525	0.0003	Sign.			
		**2**	**k**	0.2979	0.0083	3.60*10^− 6^	Sign.	0.04280	0.99097	0.98871	**k**	0.2957	0.0064	1.28*10^− 6^	Sign.	0.03484	0.99428	0.99285
			**n**	3.0110	0.3453	0.0010	Sign.				**n**	3.3313	0.3215	0.0005	Sign.			
		**3**	**k**	0.2313	0.0076	8.43*10^− 8^	Sign.	0.05657	0.98253	0.97962	**k**	0.2245	0.0042	2.74*10^− 9^	Sign.	0.03110	0.99448	0.99356
			**n**	2.9249	0.3853	0.0003	Sign.				**n**	2.7049	0.1886	7.19*10^− 6^	Sign.			
**Page**	**50**	**1**	**k**	0.0822	0.0068	5.87*10^− 6^	Sign.	0.01550	0.99849	0.99827	**k**	0.0677	0.0106	0.0007	Sign.	0.02690	0.99523	0.99443
			**n**	1.7959	0.0566	7.99*10^− 9^	Sign.				**n**	1.8631	0.1061	2.19*10^− 6^	Sign.			
		**2**	**k**	0.0196	0.0027	2.82*10^− 5^	Sign.	0.01985	0.99736	0.99710	**k**	0.0151	0.0035	0.0021	Sign.	0.03080	0.99255	0.99172
			**n**	2.1500	0.0749	6.10*10^− 11^	Sign.				**n**	2.1943	0.1235	2.56*10^− 8^	Sign.			
		**3**	**k**	0.0033	0.0010	0.0086	Sign.	0.03081	0.99411	0.99358	**k**	0.0096	0.0020	0.0005	Sign.	0.02623	0.99495	0.99449
			**n**	2.8814	0.1586	1.50*10^− 9^	Sign.				**n**	2.3025	0.1011	1.32*10^− 10^	Sign.			
	**60**	**1**	**k**	0.0566	0.0089	0.0032	Sign.	0.01881	0.99852	0.99815	**k**	0.0472	0.0042	0.0003	Sign.	0.01034	0.99954	0.99942
			**n**	2.8592	0.1559	5.21*10^− 5^	Sign.				**n**	2.8226	0.0810	4.05*10^− 6^	Sign.			
		**2**	**k**	0.0120	0.0040	0.0241	Sign.	0.03219	0.99451	0.99360	**k**	0.0143	0.0039	0.0103	Sign.	0.02756	0.99596	0.99528
			**n**	3.0150	0.2283	1.17*10^− 5^	Sign.				**n**	2.9084	0.1866	4.42*10^− 6^	Sign.			
		**3**	**k**	0.0093	0.0033	0.0225	Sign.	0.03771	0.99143	0.99036	**k**	0.0132	0.0040	0.0114	Sign.	0.03690	0.98983	0.98856
			**n**	2.7340	0.2074	1.05*10^− 6^	Sign.				**n**	2.3633	0.1687	6.52*10^− 7^	Sign.			
	**70**	**1**	**k**	0.0639	0.0243	0.0786	InSign.	0.04548	0.99144	0.98859	**k**	0.0740	0.0103	0.0055	Sign.	0.01713	0.99887	0.99850
			**n**	2.9214	0.4004	0.0053	Sign.				**n**	2.8987	0.1525	0.0003	Sign.			
		**2**	**k**	0.0261	0.0109	0.0748	InSign.	0.04280	0.99097	0.98871	**k**	0.0173	0.0067	0.0620	InSign.	0.03484	0.99428	0.99285
			**n**	3.0110	0.3453	0.0010	Sign.				**n**	3.3313	0.3215	0.0005	Sign.			
		**3**	**k**	0.0138	0.0078	0.1259	InSign.	0.05657	0.98253	0.97962	**k**	0.0176	0.0050	0.0121	Sign.	0.03110	0.99448	0.99356
			**n**	2.9249	0.3853	0.0003	Sign.				**n**	2.7049	0.1886	7.19*10^− 6^	Sign.			
**Wang-Sigh**	**50**	**1**	**b**	-0.1718	0.0155	1.07*10^− 5^	Sign.	0.05479	0.98112	0.97843	**b**	-0.1308	0.0131	5.81*10^− 5^	Sign.	0.03826	0.99034	0.98873
			**a**	0.0056	0.0024	0.0510	InSign.				**a**	-0.0016	0.0023	0.5137	InSign.			
		**2**	**b**	-0.0892	0.0106	7.83*10^− 6^	Sign.	0.05960	0.97620	0.97381	**b**	-0.0568	0.0052	1.60*10^− 6^	Sign.	0.02518	0.99502	0.99447
			**a**	-0.0006	0.0012	0.6042	InSign.				**a**	-0.0043	0.0006	8.48*10^− 5^	Sign.			
		**3**	**b**	-0.0535	0.0134	0.0021	Sign.	0.08476	0.95544	0.95139	**b**	-0.0577	0.0081	1.89*10^− 5^	Sign.	0.05133	0.98065	0.97889
			**a**	-0.0032	0.0014	0.0410	Sign.				**a**	-0.0024	0.0008	0.0143	Sign.			
	**60**	**1**	**b**	-0.2052	0.0653	0.0348	Sign.	0.11844	0.94133	0.92667	**b**	-0.1549	0.0601	0.0614	InSign.	0.10892	0.94857	0.93571
			**a**	-0.0017	0.0155	0.9155	InSign.				**a**	-0.0113	0.0142	0.4716	InSign.			
		**2**	**b**	-0.0868	0.0308	0.0304	Sign.	0.09003	0.95708	0.94993	**b**	-0.0911	0.0317	0.0283	Sign.	0.09266	0.95427	0.94665
			**a**	-0.0097	0.0053	0.1192	InSign.				**a**	-0.0088	0.0055	0.1593	InSign.			
		**3**	**b**	-0.0725	0.0178	0.0036	Sign.	0.07464	0.96643	0.96224	**b**	-0.0507	0.0057	2.02*10^− 5^	Sign.	0.02387	0.99574	0.99521
			**a**	-0.0053	0.0024	0.0592	InSign.				**a**	-0.0067	0.0008	2.46*10^− 5^	Sign.			
	**70**	**1**	**b**	-0.1489	0.0679	0.1157	InSign.	0.08980	0.96663	0.95551	**b**	-0.1639	0.0859	0.1524	InSign.	0.11368	0.95031	0.93374
			**a**	-0.0270	0.0198	0.2651	InSign.				**a**	-0.0238	0.0250	0.4121	InSign.			
		**2**	**b**	-0.0850	0.0393	0.0965	InSign.	0.07124	0.97498	0.96873	**b**	-0.0676	0.0456	0.2120	InSign.	0.08261	0.96784	0.95981
			**a**	-0.0243	0.0093	0.0591	InSign.				**a**	-0.0282	0.0108	0.0593	InSign.			
		**3**	**b**	-0.0942	0.0302	0.0205	Sign.	0.08822	0.95753	0.95045	**b**	-0.0841	0.0253	0.0158	Sign.	0.07391	0.96883	0.96364
			**a**	-0.0086	0.0052	0.1517	InSign.				**a**	-0.0094	0.0044	0.0748	InSign.			
**Weibullian**	**50**	**1**	**β**	1.7959	0.0566	7.99*10^− 9^	Sign.	0.01550	0.99849	0.99827	**β**	1.8631	0.1061	2.19*10^− 6^	Sign.	0.02690	0.99523	0.99443
			**a**	4.0204	0.0480	9.16*10^− 12^	Sign.				**a**	4.2447	0.0856	4.51*10^− 9^	Sign.			
		**2**	**β**	2.1500	0.0749	6.10*10^− 11^	Sign.	0.01985	0.99736	0.99710	**β**	2.1943	0.1235	2.56*10^− 8^	Sign.	0.03080	0.99255	0.99172
			**a**	6.2251	0.0700	7.96*10^− 16^	Sign.				**a**	6.7492	0.1156	6.40*10^− 13^	Sign.			
		**3**	**β**	2.8814	0.1586	1.50*10^− 9^	Sign.	0.03081	0.99411	0.99358	**β**	2.3025	0.1011	1.32*10^− 10^	Sign.	0.02623	0.99495	0.99449
			**a**	7.2825	0.1014	4.72*10^− 16^	Sign.				**a**	7.5288	0.0992	2.59*10^− 16^	Sign.			
	**60**	**1**	**β**	2.8592	0.1559	5.21*10^− 5^	Sign.	0.01881	0.99852	0.99815	**β**	2.8226	0.0810	4.05*10^− 6^	Sign.	0.01034	0.99954	0.99942
			**a**	2.7301	0.0380	2.25*10^− 7^	Sign.				**a**	2.9493	0.0218	1.80*10^− 8^	Sign.			
		**2**	**β**	3.0150	0.2283	1.17*10^− 5^	Sign.	0.03219	0.99451	0.99360	**β**	2.9084	0.1866	4.42*10^− 6^	Sign.	0.02756	0.99596	0.99528
			**a**	4.3314	0.0798	2.63*10^− 9^	Sign.				**a**	4.3030	0.0693	1.18*10^− 9^	Sign.			
		**3**	**β**	2.7340	0.2074	1.05*10^− 6^	Sign.	0.03771	0.99143	0.99036	**β**	2.3633	0.1687	6.52*10^− 7^	Sign.	0.03690	0.98983	0.98856
			**a**	5.5419	0.1111	2.89*10^− 11^	Sign.				**a**	6.2341	0.1278	3.46*10^− 11^	Sign.			
	**70**	**1**	**β**	2.9214	0.4004	0.0053	Sign.	0.04548	0.99144	0.98859	**β**	2.8987	0.1525	0.0003	Sign.	0.01713	0.99887	0.99850
			**a**	2.5644	0.0881	8.91*10^− 5^	Sign.				**a**	2.4550	0.0326	5.17*10^− 6^	Sign.			
		**2**	**β**	3.0110	0.3453	0.0010	Sign.	0.04280	0.99097	0.98871	**β**	3.3313	0.3215	0.0005	Sign.	0.03484	0.99428	0.99285
			**a**	3.3564	0.0936	3.60*10^− 6^	Sign.				**a**	3.3813	0.0727	1.28*10^− 6^	Sign.			
		**3**	**β**	2.9249	0.3853	0.0003	Sign.	0.05657	0.98253	0.97962	**β**	2.7049	0.1886	7.19*10^− 6^	Sign.	0.03110	0.99448	0.99356
			**a**	4.3233	0.1423	8.43*10^− 8^	Sign.				**a**	4.4536	0.0827	2.74*10^− 9^	Sign.			
**Weibullian (I)**	**50**	**1**	**n**	1.7959	0.0566	7.99*10^− 9^	Sign.	0.01550	0.99849	0.99827	**n**	1.8631	0.1061	2.19*10^− 6^	Sign.	0.02690	0.99523	0.99443
			**δ**	6.3967	0.1188	2.00*10^− 10^	Sign.				**δ**	6.6415	0.2200	8.76*10^− 8^	Sign.			
		**2**	**n**	2.1500	0.0749	6.10*10^− 11^	Sign.	0.01985	0.99736	0.99710	**n**	2.1943	0.1235	2.56*10^− 8^	Sign.	0.03080	0.99255	0.99172
			**δ**	9.1752	0.1637	7.94*10^− 14^	Sign.				**δ**	9.8700	0.2864	7.21*10^− 11^	Sign.			
		**3**	**n**	2.8814	0.1586	1.50*10^− 9^	Sign.	0.03081	0.99411	0.99358	**n**	2.3025	0.1011	1.32*10^− 10^	Sign.	0.02623	0.99495	0.99449
			**δ**	9.7272	0.2154	7.68*10^− 14^	Sign.				**δ**	10.8152	0.2317	5.34*10^− 14^	Sign.			
	**60**	**1**	**n**	2.8592	0.1559	5.21*10^− 5^	Sign.	0.01881	0.99852	0.99815	**n**	2.8226	0.0810	4.05*10^− 6^	Sign.	0.01034	0.99954	0.99942
			**δ**	3.6548	0.0806	1.42*10^− 6^	Sign.				**δ**	3.9632	0.0465	1.14*10^− 7^	Sign.			
		**2**	**n**	3.0150	0.2283	1.17*10^− 5^	Sign.	0.03219	0.99451	0.99360	**n**	2.9084	0.1866	4.42*10^− 6^	Sign.	0.02756	0.99596	0.99528
			**δ**	5.7117	0.1674	4.23*10^− 8^	Sign.				**δ**	5.7320	0.1468	1.88*10^− 8^	Sign.			
		**3**	**n**	2.7340	0.2074	1.05*10^− 6^	Sign.	0.03771	0.99143	0.99036	**n**	2.3633	0.1687	6.52*10^− 7^	Sign.	0.03690	0.98983	0.98856
			**δ**	7.5187	0.2399	1.17*10^− 9^	Sign.				**δ**	8.8724	0.3065	2.19*10^− 9^	Sign.			
	**70**	**1**	**n**	2.9214	0.4004	0.0053	Sign.	0.04548	0.99144	0.98859	**n**	2.8987	0.1525	0.0003	Sign.	0.01713	0.99887	0.99850
			**δ**	3.4117	0.1859	0.0004	Sign.				**δ**	3.2735	0.0690	2.06*10^− 5^	Sign.			
		**2**	**n**	3.0110	0.3453	0.0010	Sign.	0.04280	0.99097	0.98871	**n**	3.3313	0.3215	0.0005	Sign.	0.03484	0.99428	0.99285
			**δ**	4.4276	0.1968	2.31*10^− 5^	Sign.				**δ**	4.3432	0.1490	8.25*10^− 6^	Sign.			
		**3**	**n**	2.9249	0.3853	0.0003	Sign.	0.05657	0.98253	0.97962	**n**	2.7049	0.1886	7.19*10^− 6^	Sign.	0.03110	0.99448	0.99356
			**δ**	5.7498	0.3008	1.33*10^− 6^	Sign.				**δ**	6.0621	0.1794	4.47*10^− 8^	Sign.			

MMs is the mathematical models; DAT is the drying air temperature, ℃; LT is the layer thickness, cm; OD is the oven drying system; HSDS is the hybrid solar drying system; k is the drying constant, h^− 1^; a, b, c, d, and n are the models constants, dimensionless; R^2^ is the coefficient of determination; RSME is the root mean square error.

**Table 5:** Best mathematical models for OD and HSDS under different drying temperature and layers thicknesses for drying ESML.

OD			HSD		
Weibullian (I) at DAT50LT1			Midilli at DAT60LT1		
RMSE	R^2^	R^2^_adj._	RMSE	R^2^	R^2^_**adj.**_
0.01550	0.99849	0.99827	0.01020	0.99977	0.99944

### Thermodynamic properties

Table 6 presents the thermodynamic parameters (enthalpy, entropy, and Gibbs free energy) of OD and HSDS for ESML at various drying temperatures and layer thicknesses. The tabulated data indicated the values of enthalpy, entropy, and Gibbs free energy at various drying temperatures. As drying temperature rises, enthalpy and entropy diminish, however Gibbs free energy escalates. Enthalpy pertains to the energy required to eliminate the water associated with dry matter during drying, thereby decreasing with elevated drying temperatures^112,113^. Low enthalpy values at reduced temperatures signify an increased energy demand for the drying of tamarind seeds; analogous behavior was noted in the drying processes of Baru fruits examined by Resende et al.^114^ Bode pepper grains investigated by Rodovalho et al.^115^and tamarind seeds analyzed by Ferreira Junior et al.^116^. Entropy values (Table 6) diminish as drying temperature rises, as elevated temperatures significantly enhance the excitation of the product’s water molecules relative to lower temperatures, hence reducing the order of the water-product system^116,117^. Entropy, a thermodynamic property linked to the level of disorder between water and the product^116,118^ exhibits decreasing values with rising drying temperatures. Negative entropy values are ascribed to the presence of chemical adsorption and/or structural alterations of the adsorbent^119^. Positive Gibbs free energy values (Table 6) signify an endergonic reaction, necessitating the addition of energy to the air for the product’s drying process to transpire 112. Similar trends have been noted in studies^113,120,121^.

**Table 6:** Thermodynamic properties of OD and HSDS for ESML under different drying temperatures and layers thicknesses.

Drying system	DAT, ºC	LT, cm	Enthalpy (ΔH), kJ mol^− 1^	Entropy (ΔS), kJ mol^− 1^ K^− 1^	Gibbs free energy (ΔG), kJ mol^− 1^
OD	50	1	29.27	-0.175	85.78
		2	31.96	-0.192	93.85
		3	24.99	-0.176	81.82
	60	1	29.18	-0.175	87.53
		2	31.87	-0.192	95.77
		3	24.91	-0.176	83.58
	70	1	29.10	-0.175	89.28
		2	31.79	-0.192	97.69
		3	24.83	-0.176	85.35
HSD	50	1	29.99	-0.177	87.05
		2	39.22	-0.213	107.95
		3	24.67	-0.173	80.68
	60	1	29.91	-0.177	88.82
		2	39.13	-0.213	110.08
		3	24.58	-0.174	82.41
	70	1	29.83	-0.177	90.58
		2	39.05	-0.213	112.21
		3	24.50	-0.174	84.15

## Conclusion

In this research, Egyptian sweet marjoram leaves (ESML) were dried using a hybrid solar drying system (HSDS) at three different temperatures of 50, 60, and 70 °C and three different layer thicknesses of 1, 2, and 3 cm. Then, the OD was used to compare the performance of the drying process with the HSDS in terms of drying characteristics, effective moisture diffusivity (EMD), mathematical modeling, activation energy, and thermodynamic properties. During the drying process, the MC reduced readings from an average MC of 77.50% to equilibrium moisture content of 1.95% and 2.15% (w.b.) for OD and HSDS, respectively. On the other hand, raising the air temperature from 50 to 70 °C leads to a reduction in the time required for the finished product to dry by about 100%, 120%, and 71.4% for layer thicknesses of 1 cm, 2 cm, and 3 cm, respectively, for OD, while the drying time was reduced by about 75%, 100%, and 71.4% for layer thicknesses of 1 cm, 2 cm, and 3 cm, respectively, for HSDS. Additionally, the EMD increases with increasing temperature and layer thickness. And the highest EMDs were 14.1 × 10^− 9^ m^2^ s^− 1^, and 12 × 10^− 9^ m^2^ s^− 1^ for the OD and HSDS, respectively. They were observed at a 70 °C drying temperature and a 3 cm layer thickness. The activation energy values for the thickness of 1.0, 2.0, and 3.0 cm ranged between 27.68 and 34.64 kJ.mol-1 and 27.35–41.9 kJ.mol-1, respectively. In addition, Weibullian (I) and Midilli were the best mathematical models to show how ESML dried for OD and HSDS, respectively. Moreover, results showed that with the increase in drying temperature, enthalpy and entropy decreased, while Gibbs free energy increased.

### Future work

Future studies should focus on investigating the comparative effects of oven drying and hybrid solar drying on the physical, chemical, and quality attributes of marjoram. This includes assessing changes in essential oil content, color, texture, antioxidant activity, and overall sensory properties to determine the most efficient and quality-preserving drying method.

### Practical applications

Dried Egyptian sweet marjoram leaves are widely used for their aromatic, medicinal, and preservative properties. They enhance food flavor, support digestive and respiratory health in herbal remedies, and exhibit antioxidant and antimicrobial effects. Their practical applications span culinary, pharmaceutical, and cosmetic industries, offering natural, functional ingredients with extended shelf life.

## Data Availability

All data is provided within the article.
